# Terahertz Photons Ameliorate Traumatic Brain Injury and Reduce Neuroinflammation via the Cyclic Adenosine Monophosphate Signaling Pathway

**DOI:** 10.34133/research.1468

**Published:** 2026-10-27

**Authors:** Jingzhou Liu, Xin Zhao, Yuan Zhong, Junkai Yin, Hongwei Wu, Tingrong Zhang, Yun Yu, Rundong Jiang, Yue Wang, Chao Chang

**Affiliations:** ^1^ Innovation Laboratory of Terahertz Biophysics, National Innovation Institute of Defense Technology, Beijing, China.; ^2^ National Key Laboratory of Brain-Computer Interface Technology, Beijing, China.; ^3^ Department of Engineering Physics, Tsinghua University, Beijing, China.; ^4^ School of Life Science and Technology, Xi’an Jiaotong University, Xi’an, Shaanxi, China.; ^5^ School of Physics, Peking University, Beijing, China.

## Abstract

Traumatic brain injury (TBI) has the highest incidence among common neurological disorders and remains the leading cause of mortality and disability among all kinds of injuries, yet specific therapies for TBI are still unavailable. In the present study, we found that short-term administration of terahertz (THz) photons during the acute phase of TBI ameliorated brain damage and cognitive deficits. THz photons induced a reduced population of microglia and an increased number of anti-inflammatory phenotypes of microglia. In vivo and in vitro validation further revealed that THz photons inhibit inflammasome activation. Blocking adenylyl cyclase type 9 (AC9) in microglia attenuated the beneficial effects of THz irradiation, indicating that THz-irradiation-mediated inhibition of inflammasome activation involves the AC9/cyclic adenosine monophosphate/NLR and pyrin domain-containing protein 3 signaling pathway. Compared with drug therapy, THz photon technology offers noninvasiveness, accurate tissue targeting, and on-demand application and removal. In conclusion, this study established a THz-photon-based therapeutic paradigm, highlighting its potential as a therapy for TBI and potentially other forms of acute brain injury.

## Introduction

Traumatic brain injury (TBI) represents the most prevalent neurological disorder globally and remains the leading cause of injury-related mortality and disability [1], which often results from road traffic accidents, falls, sports injuries, or blast injuries suffered in military conflict. It is estimated that 55 to 74 million individuals worldwide suffered TBI each year [2,3]. TBI includes primary brain injury and secondary brain injury. Primary brain injury is known as primary external impact and is not amenable to intervention. Secondary brain injury occurs in minutes after the primary injury and involves a molecular and cellular cascade that leads to further neuronal loss and functional impairment [4]. Therapeutic intervention for TBI has been focused on mitigating the progressive degeneration and neurological dysfunction during the secondary brain injury, but clinical translation remains a challenge.

Neuroinflammation plays a pivotal role in the pathological progression of TBI. Activation of immune cells results in chronic neuroinflammation, which ultimately gives rise to neurodegeneration by pro-inflammatory cytokines, oxidative stress, and proteases produced by activated microglia and exogenous immune cells [5]. For example, activated microglia display a spectrum of activation states and maintain an ability to transition between functional phenotypes. Classical classification categorizes activated microglia into 2 polarization states: the pro-inflammatory M1 phenotype and anti-inflammatory M2 phenotype [6]. Many research studies focus on the regulation of microglia polarization to increase the anti-inflammatory function of M2-like microglia by various signaling pathways, such as the nucleotide-binding oligomerization domain (NOD)-like receptor (NLR) pathway [7–9]. The core component of the NLR pathway is the inflammasome complex, which converts pro-interleukin-1β (pro-IL-1β) and pro-interleukin-18 (pro-IL-18) into active pro-inflammatory cytokines and leads to caspase-1 activation, which subsequently cleaves gasdermin D (GSDMD) to generate a pore-forming N-terminal fragment in the cell membrane [9,10]. These pores mediate the release of pro-inflammatory cytokines, which then interact with neighboring cells and initiate an inflammatory cascade reaction [9]. Overall, targeting neuroinflammation is a reasonable strategy for reducing the burden of TBI. Unfortunately, persistent neuroinflammation and neurodegeneration fall into a vicious circle, for which no effective therapeutic strategy is currently available to break.

Located in the spectral range between microwaves and infrared light, terahertz (THz; 10^12^ Hz) photons possess low photon energy and negligible ionization effects and can thus maintain genomic integrity [11–13]. THz photons at specific frequencies have been shown to exert specific nonthermal regulatory effects on ion channel permeability [14], molecular isomerization [15], and chromatin accessibility [16] through its resonant interaction with biomolecules [17]. Therefore, THz photons have provided a new approach for biosensing and nondrug biomodulation. Evidence shows that THz photons with a specific frequency can regulate neuronal activity [18], synaptic signaling [19], neuronal growth, and dendritic spine formation [13]. Moreover, THz photons can be absorbed by glutamate receptors and enhance the binding of glutamate molecules to glutamate receptors [20]. Administration of THz radiation to the hippocampal CA3 region restores cognitive function in post-traumatic stress disorder mice [20]. THz radiation also attenuates the binding of glutamate molecules to glutamate ionotropic receptor AMPA type subunit 2 (GluA2), exerting nonthermal effects on neuronal activity, which further alleviates pain and comorbid anxiety-like behaviors [12]. Although the above evidence illustrates the potential of THz photons in neuronal protection and neurological diseases, it remains unknown whether THz photons are effective in the management of TBI, in which neuroinflammation plays an important role.

This study developed a 34.5-THz-based, noninvasive, and drug-free approach aimed at mitigating TBI. THz photons improved cognitive function, attenuated neuropathology, and reduced chronic neuroinflammation in TBI mice. The therapeutic effect is mediated by THz-induced inhibition of NLR and pyrin domain-containing protein 3 (NLRP3) inflammasome complex activation in microglia. Further analysis revealed that THz photons promote cyclic adenosine monophosphate (cAMP) production in a manner associated with adenylyl cyclase type 9 (AC9), thereby inhibiting the NLRP3 inflammasome. The protective effects of THz photons were reduced by blocking AC9 on microglia. In contrast to drugs that block AC9 or NLRP3, THz photons can directly irradiate localized brain tissue, and this approach possesses a potential technical advantage in that it enables physical targeting, thus minimizing off-target adverse effects. Moreover, THz photons can be applied or withdrawn at will, enabling accurate temporal regulation of therapy with reversible action, thereby exhibiting marked advantages in the treatment of acute brain injury.

## Results

### THz photons attenuate TBI-induced cognitive deficit

To investigate the therapeutic efficacy of THz photons on TBI, we utilized the well-established and highly reproducible controlled cortical impact (CCI) model and developed an irradiation system designed to deliver THz photons precisely to the injured brain tissue, with irradiation performed at 1, 24, and 48 postinjury (10 min per session) (Fig. 1A). The Morris water maze (MWM) and novel object location (NOL) tests were performed to investigate the effect of THz photons on the cognitive function of TBI mice (Fig. 1A). The successful replication of the TBI model was confirmed by magnetic resonance imaging (MRI) at 3 d postinjury (dpi), which demonstrated characteristic edema and contusion at the impact site (Fig. 1B). The applied power densities were 4 and 7 mW/mm^2^ for the respective irradiation groups. In the MWM test, TBI mice treated with 7 mW/mm^2^ irradiation exhibited a remarkably shortened escape latency and increased number of target crossings and time in target quadrant compared to the TBI controls, while the TBI+4 mW/mm^2^ group showed no statistical improvement in these parameters (Fig. 1D). Moreover, no statistically significant differences were observed in total swimming distance or mean speed between the TBI group and the TBI+4 mW/mm^2^ or TBI+7 mW/mm^2^ group, indicating that THz irradiation does not exert a substantial effect on motor performance (Fig. S1A). These findings suggest that THz photons with 7 mW/mm^2^ enhance spatial memory after TBI. In the NOL test, the TBI+7 mW/mm^2^ group exhibited a significantly higher location recognition index compared with the TBI group, while the TBI+4 mW/mm^2^ group showed no significant improvement (Fig. 1F). Additionally, the open-field test was conducted on the Sham, TBI, and TBI+7 mW/mm^2^ groups. There were no significant differences in center entries, time in the center, or total distance across the 3 groups, suggesting that the TBI procedure and THz exposure in this study do not influence locomotor activity or anxiety-like responses in mice (Fig. S1B). In conclusion, these data demonstrate that 7 mW/mm^2^ THz irradiation improves spatial learning and cognitive outcomes in TBI mice, with a greater effect than 4 mW/mm^2^ THz irradiation. Based on these findings, 7 mW/mm^2^ was therefore selected as the standard irradiation parameter for the remainder of the study.

**Fig. 1.**
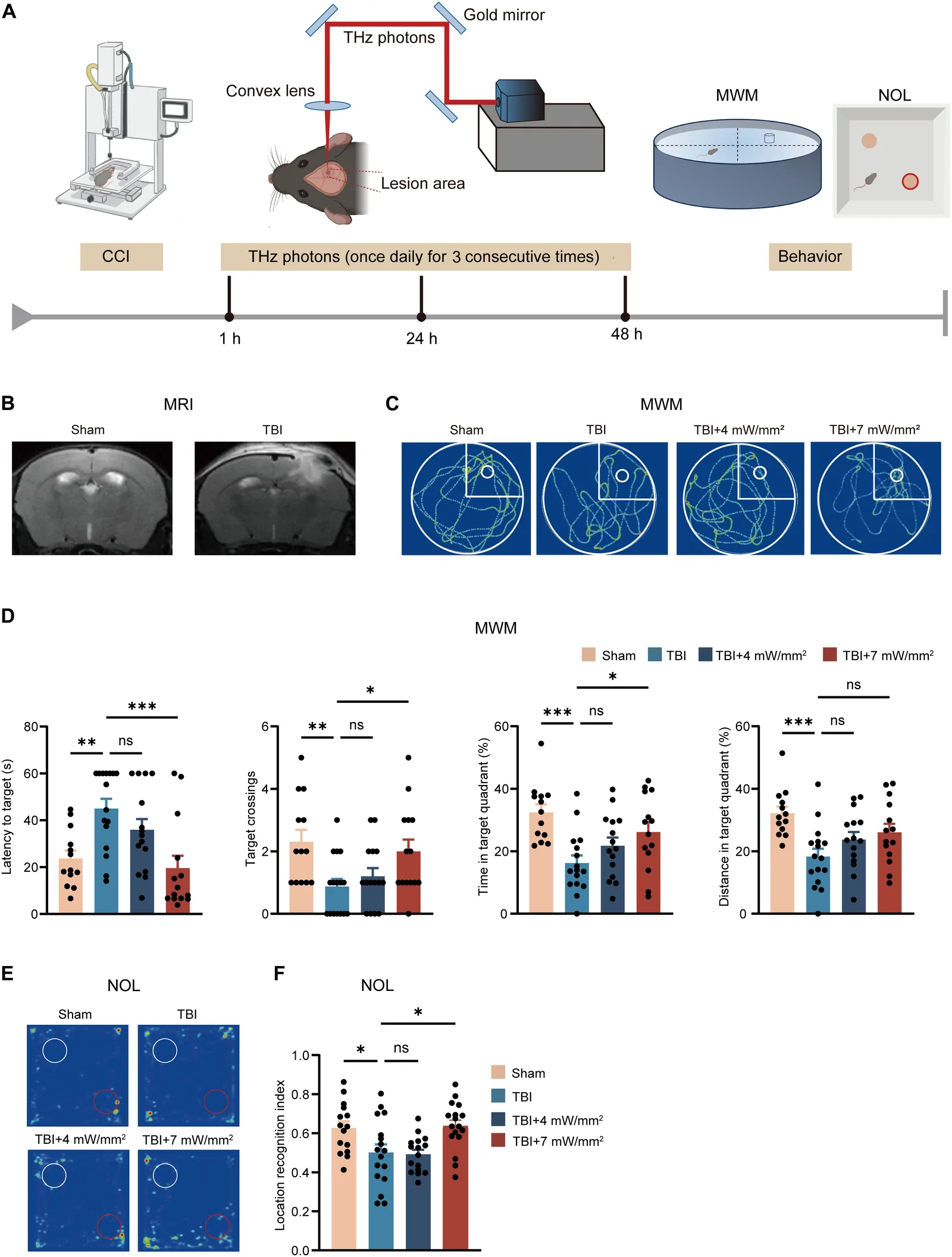
Terahertz (THz) photons improve spatial learning and memory outcomes after traumatic brain injury (TBI). (A) Schematic diagram of the experimental timeline for controlled cortical impact model construction, THz photon exposure, and behavior tests. (B) Magnetic resonance imaging for the Sham and TBI groups. (C) Representative animal tracks in the Morris water maze (MWM) for the Sham, TBI, TBI+4 mW/mm^2^, and TBI+7 mW/mm^2^ groups. (D) Latency to target, target crossings, percentage time in target quadrant, and percentage distance in target quadrant of the MWM. Target crossings were analyzed using the Kruskal–Wallis test with Dunn post hoc analysis; other data were analyzed by one-way analysis of variance (ANOVA) followed by a Bonferroni post hoc test. Sham (*n* = 13), TBI (*n* = 16), TBI+4 mW/mm^2^ (*n* = 15), and TBI+7 mW/mm^2^ (*n* = 14). (E) Representative heatmaps in novel object location (NOL) for the Sham, TBI, TBI+4 mW/mm^2^, and TBI+7 mW/mm^2^ groups. The red circle indicates the new location. (F) Location recognition index of NOL. Sham (*n* = 15), TBI (*n* = 17), TBI+4 mW/mm^2^ (*n* = 16), and TBI+7 mW/mm^2^ (*n* = 17). One-way ANOVA followed by a Bonferroni post hoc test was used. Data are expressed as mean ± standard error of the mean (SEM). ns, not significant; *P < 0.05; **P < 0.01; ***P < 0.001.

### THz photons ameliorate TBI neuropathology

TBI induces tissue loss, cell death, and neuronal functional impairment [21]. Given these characteristic neuropathological changes, we evaluated the effects of THz photons on these key outcomes (Fig. 2A). First, we measured parenchymal lesion volume in the TBI and TBI+THz groups at 21 dpi by Nissl staining. The TBI+THz group exhibited a marked reduction in lesion volume compared with the TBI group (Fig. 2B). We also examined cell death using terminal deoxynucleotidyl transferase-mediated dUTP nick-end labeling (TUNEL) staining at 7 dpi. TBI induced a marked increase in TUNEL-positive cells compared with the Sham group, while THz photons substantially attenuated this increase relative to TBI controls (Fig. 2C). To determine whether THz preserves the synaptic structure following TBI, we analyzed neuronal morphology via Golgi staining at 7 dpi. THz photons increased dendritic spine density by 69.8% compared with TBI controls, restoring this parameter to levels comparable to those of the Sham group (Fig. 2D). Moreover, Sholl analysis for neuronal architecture showed that THz treatment increased neuronal branching complexity, yet the value was still reduced compared with that of sham animals (Fig. 2E). This mismatch in structural recovery may be attributed to their different remodeling rates post-TBI: dendritic spines possess high plasticity and recover rapidly under THz intervention, while the reconstruction of complex dendritic branches is a slower process that cannot be fully reversed within the experimental period [22]. In addition, THz photons did not affect the cerebral blood flow in TBI mice (Fig. S2). Together, these data demonstrate that the behavioral improvements observed in THz-treated mice are associated with enhanced tissue integrity and synaptic complexity.

**Fig. 2.**
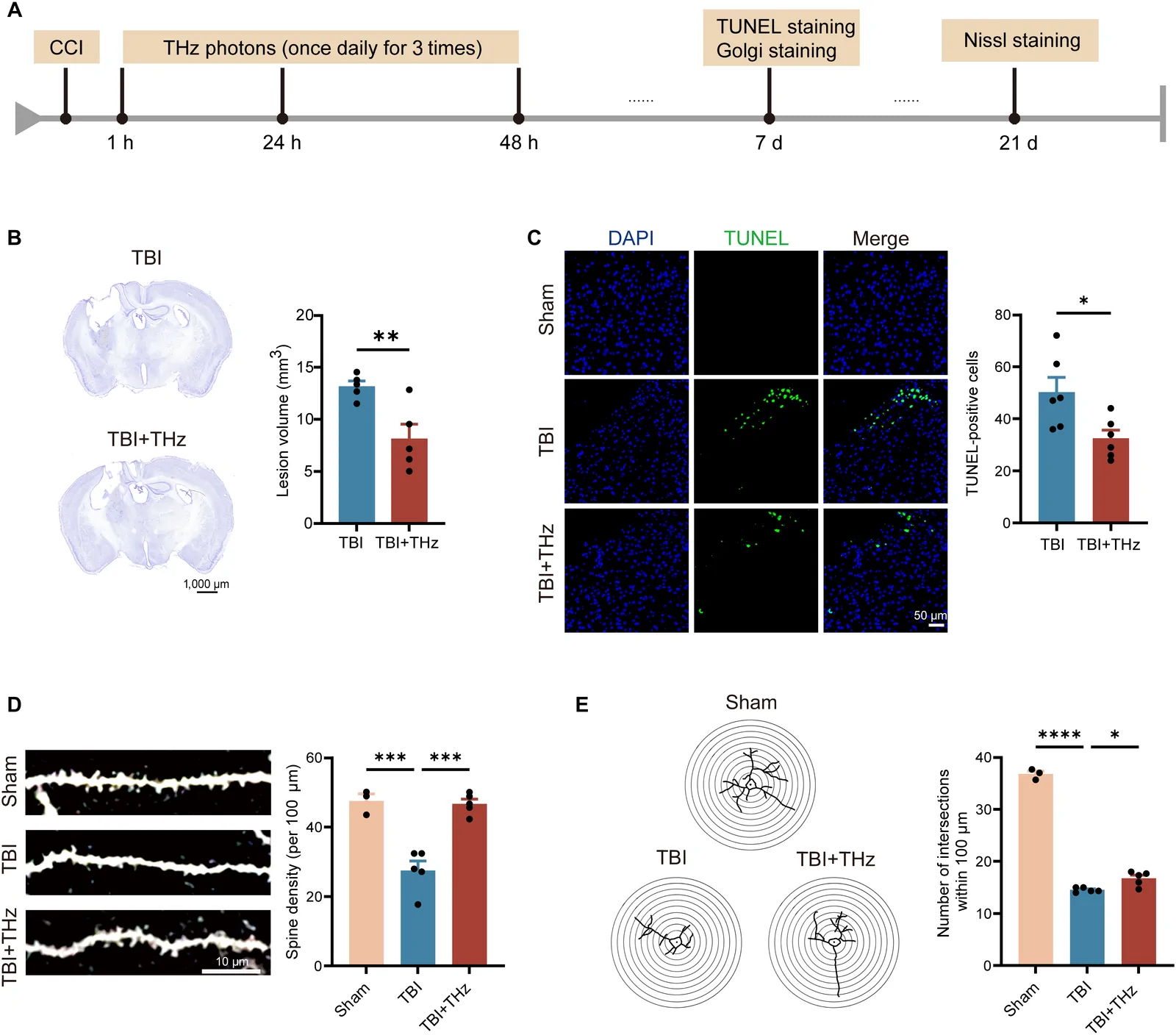
Terahertz (THz) photons ameliorate neuropathological outcomes in a traumatic brain injury (TBI) model. (A) Schematic of the experimental timeline. (B) Lesion volume from Nissl staining brain sections at 21 d postinjury (dpi). TBI and TBI+THz (*n* = 5). Scale bars = 1,000 μm. (C) Terminal deoxynucleotidyl transferase-mediated dUTP nick-end labeling (TUNEL) staining at 7 dpi (4′,6-diamidino-2-phenylindole [DAPI], blue; TUNEL, green). Scale bars = 50 μm. TBI and TBI+THz (*n* = 6). (D) Representative images of Golgi-stained dendritic spines and quantitative analysis of spine density. Scale bars = 10 μm. Sham (*n* = 3); TBI and TBI+THz (*n* = 5). (E) The representative Sholl analysis and quantitative analysis of the number of intersections per 100 μm. Sham (*n* = 3); TBI and TBI+THz (*n* = 5). (B) and (C) were analyzed by a 2-sided, unpaired Student *t* test. (D) and (E) were analyzed by one-way analysis of variance (ANOVA) with a Bonferroni post hoc test. Data are shown as mean ± standard error of the mean (SEM). *P < 0.05; **P < 0.01; ***P < 0.001; ****P < 0.0001.

### THz photons alleviate neuroinflammation after TBI

TBI leads to a cascade of immune events mediated by microglia, astrocytes, and infiltrated immune cells [23–25]. We first performed immunofluorescence (IF) staining of ionized calcium-binding adapter molecule 1 (Iba1)^+^ myeloid cells at 7 dpi. The THz-treated group showed decreased Iba1^+^ cells compared to the TBI group, indicating a reduction of microglia or macrophage activation (Fig. 3A and B). We further performed reverse transcription quantitative polymerase chain reaction (RT-qPCR) and IF staining. The characteristic markers CD16 and CD206 or arginase-1 (Arg1) were employed to identify M1 (pro-inflammatory) and M2 (anti-inflammatory) microglia or macrophages, respectively [26]. In the peri-lesional brain tissue, there was no significant difference in the CD16 messenger RNA (mRNA) level between the 2 groups, while the CD206 mRNA level remained higher in the THz-treated group than in the TBI group (Fig. 3C). In line with these observations, IF staining revealed that there was no significant difference in CD16^+^ cells between the 2 groups (Fig. 3D and E), while Arg1^+^ cells remained significantly higher in the THz mice than in the TBI mice (Fig. 3F and G). Moreover, despite a reduction in the absolute number of Iba1^+^ cells, the absolute number of Arg1^+^ cells increased, while the absolute number of CD16^+^ cells decreased (Tables S1 and S2). These results suggest that THz photons promote a beneficial immunomodulatory effect after TBI, characterized by the enhancement of anti-inflammatory M2-like microglia or macrophages. Neutrophils, one of the major infiltrated immune cells, are recruited to the injury site by chemokines (e.g., C-X-C motif chemokine ligand 1 [CXCL1]) secreted by microglia and astrocytes after TBI [27,28]. We observed the THz-treated group exhibited down-regulation of CXCL1, compared with the TBI group (Fig. 3H). Next, flow cytometric analysis at 7 dpi revealed that TBI mice receiving THz irradiation had a lower percentage of neutrophils compared to TBI mice, albeit without statistical significance (Fig. 3I and J). We also quantified the proportion of neutrophils at 3 and 14 dpi, which exhibited similar distribution patterns as that observed at 7 dpi. No significant differences were detected between the TBI and TBI+THz groups (Fig. S3). These findings suggest that neutrophils may not be the direct target of THz-mediated regulation. No significant differences were observed in the percentages of macrophages, CD4^+^ T cells, CD8^+^ T cells, and regulatory T cells (Tregs) between the TBI group and the TBI+THz group (Fig. S4). In conclusion, these results suggest that THz photons primarily regulate the activation and polarization of microglia, thus alleviating neuroinflammation after TBI.

**Fig. 3.**
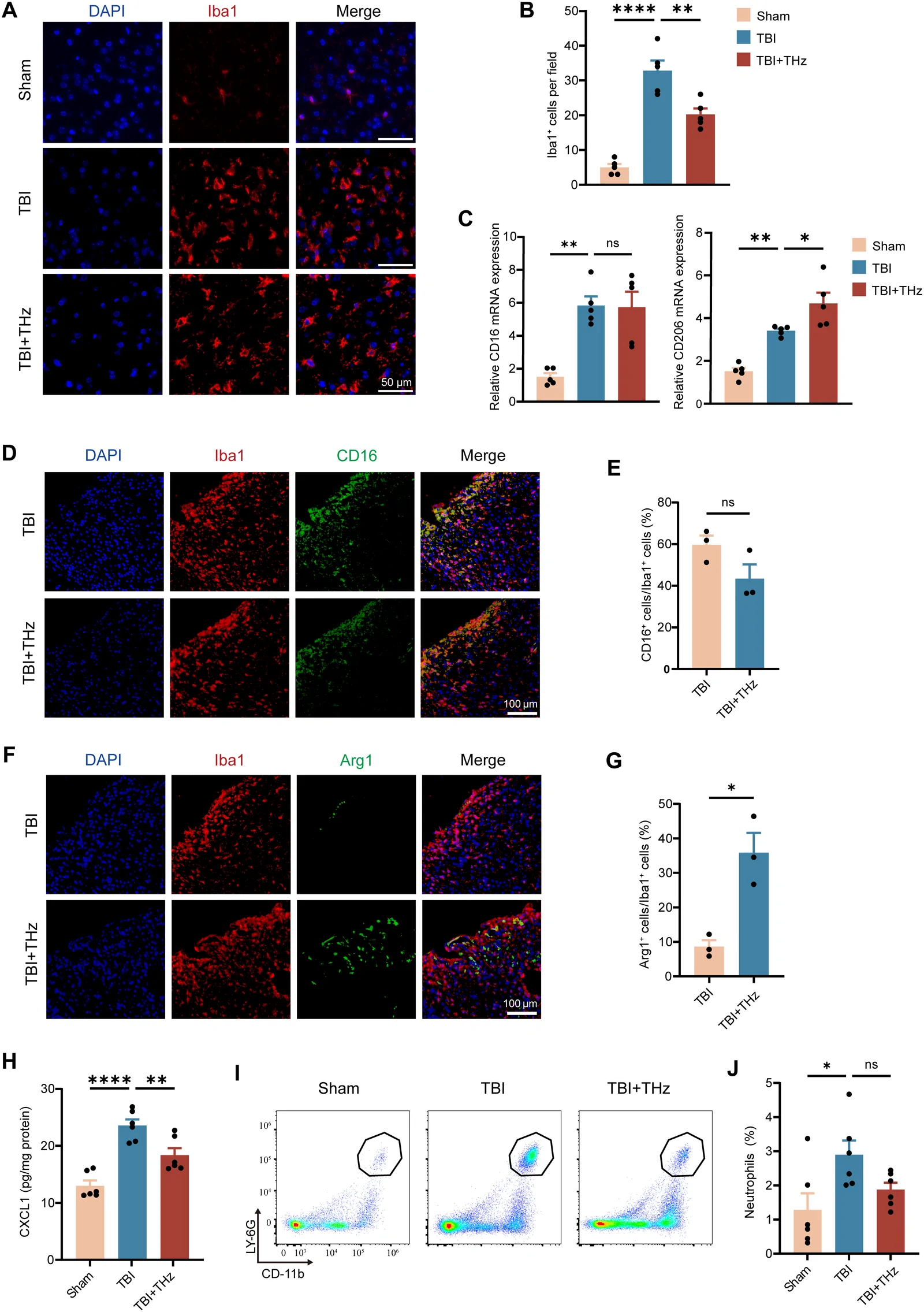
Terahertz (THz) photons inhibit neuroinflammation in a traumatic brain injury (TBI) model. (A) Immunofluorescence images for ionized calcium-binding adapter molecule 1 (Iba1) in the Sham, TBI, and TBI+THz groups. Scale bar = 50 μm. (B) Quantitative analysis of Iba1^+^ cells (*n* = 5). (C) Quantitative analysis for relative messenger RNA (mRNA) expression of CD16 and CD206 by reverse transcription quantitative polymerase chain reaction (RT-qPCR) (*n* = 5). (D) Immunofluorescence images for Iba1 and CD16 in the TBI and TBI+THz groups. Scale bar = 100 μm. (E) Quantitative analysis of CD16^+^ cells/Iba1^+^ cells (*n* = 3). (F) Immunofluorescence images for Iba1 and arginase-1 (Arg1) in the TBI and TBI+THz groups. Scale bar = 100 μm. (G) Quantitative analysis of Arg1^+^ cells/Iba1^+^ cells (*n* = 3). (H) Quantitative analysis of the C-X-C motif chemokine ligand 1 (CXCL1) concentration in the lesion region (*n* = 6). (I) Flow cytometry scatterplots of neutrophils isolated from the lesion area at 7 d postinjury (dpi). (J) Quantitative analysis of the neutrophil proportion of the Sham, TBI, and TBI+THz groups (*n* = 6). (B), (C), (H), and (J) were analyzed by one-way analysis of variance (ANOVA) with a Bonferroni post hoc test. (E) and (G) were analyzed by a 2-sided, unpaired Student *t* test. Data are shown as mean ± standard error of the mean (SEM). ns, not significant; *P < 0.05; **P < 0.01; ****P < 0.0001.

### THz photons regulate post-TBI immunity via the up-regulation of cAMP and down-regulation of the NLR signaling pathway

To uncover the molecular basis for the therapeutic effects of THz photons on neuroinflammation and neuropathology, we performed RNA sequencing and proteomic analyses to compare gene and protein expression patterns between the TBI group and the TBI+THz group. RNA sequencing profiling revealed 1,096 up-regulated and 1,173 down-regulated genes in the TBI+THz group versus the TBI group (Fig. S5). Among these, several differentially expressed genes down-regulated in the TBI+THz group were involved in immune responses, including *Cd33*, *Gbp6*, *Teme173*, *Pik3cg*, *Ifih1*, and *Cflar* (Fig. 4A). Gene Ontology (GO) enrichment analysis showed that THz photon treatment affected biological processes related to immune response, cell leading edge, and distal axon compared with TBI controls (Fig. 4B). Subsequent Kyoto Encyclopedia of Genes and Genomes (KEGG) analysis highlighted the cAMP signaling pathway as the most enriched up-regulated pathway (Fig. 4C) and the NLR signaling pathway as the most enriched down-regulated pathway (Fig. 4D). Parallel proteomic GO analysis highlighted lyase activity, phosphoprotein phosphatase activity, and integral component of membrane (Fig. 4E). Notably, proteomic KEGG analysis reinforced the marked enrichment of the NLR signaling pathway, which was also identified in our RNA sequencing data (Fig. 4F). Given that this pathway is known to aggravate disease progression, particularly in the acute and subacute phases of TBI [9], its coordinated down-regulation at both the transcriptional and protein levels identify a candidate pathway of THz treatment.

**Fig. 4.**
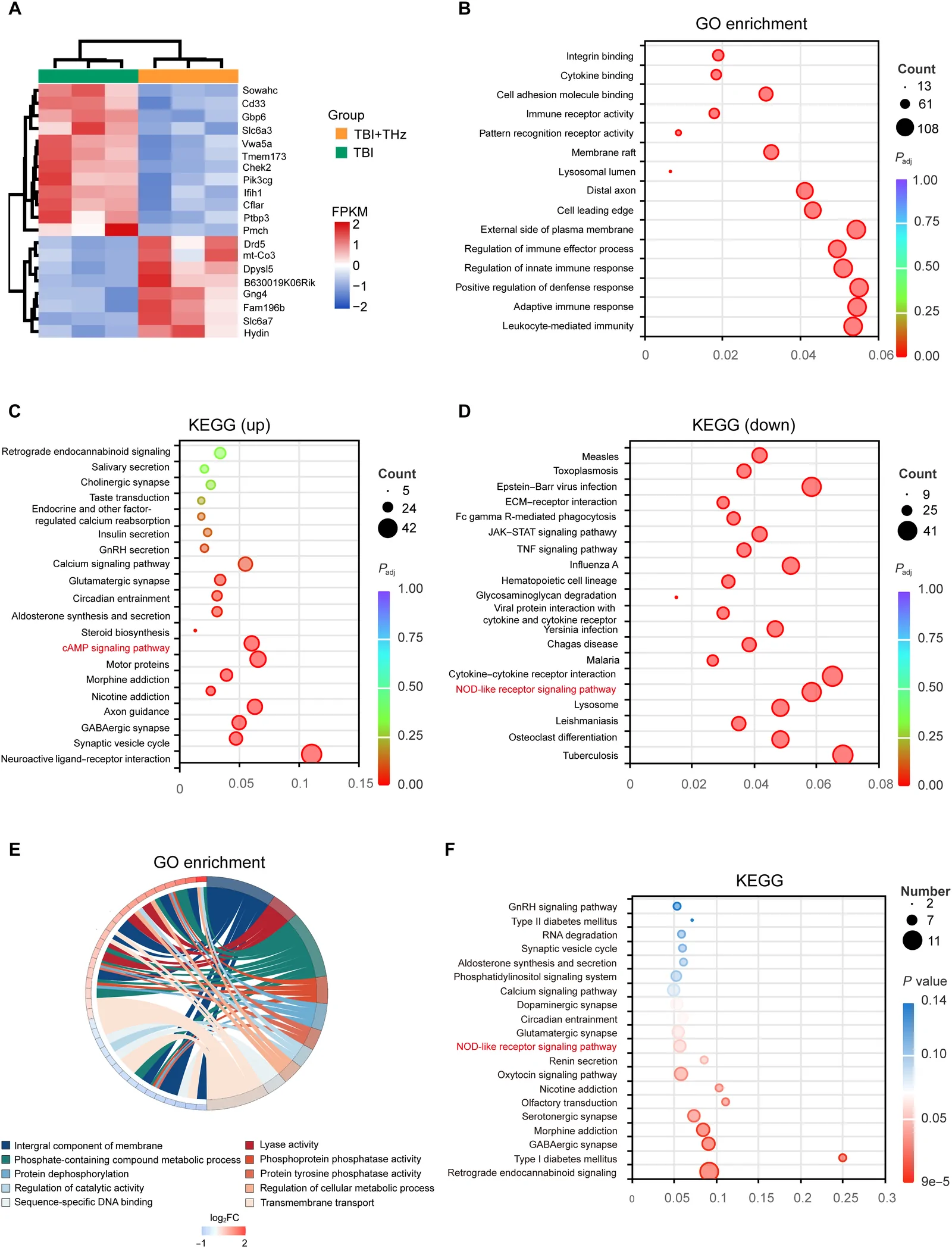
Terahertz (THz) photons regulate the cyclic adenosine monophosphate (cAMP) and nucleotide-binding oligomerization domain (NOD)-like receptor signaling pathway based on RNA sequencing and proteomics. (A) Heatmap of differentially expressed genes (DEGs). (B) Gene Ontology (GO) enrichment analysis of DEGs. (C) Kyoto Encyclopedia of Genes and Genomes (KEGG) enrichment (up) analysis of DEGs. (D) KEGG enrichment (down) analysis of DEGs. (E) GO enrichment analysis based on proteomics. (F) KEGG enrichment analysis based on proteomics. All results are represented as TBI+THz vs TBI. TBI, traumatic brain injury.

### THz photons inhibit NLRP3 inflammasome activation by increasing the cAMP level

The NLR signaling pathway senses intracellular danger signals and orchestrates inflammatory responses, largely through the activation of its most characterized effector, the NLRP3 inflammasome [9]. Upon activation, NLRP3 assembles with apoptosis-associated speck-like protein containing a CARD (ASC) and pro-caspase-1 to form the inflammasome complex. Therefore, to validate the omics findings, we assessed the protein expression of key inflammasome components in this pathway, including NLRP3 and ASC. At 7 dpi, western blot analysis revealed that THz photons decreased the expression of NLRP3 and ASC by 25.3% and 29.3%, respectively, compared to TBI controls, indicating that THz photon treatment effectively suppresses the activation of the NLRP3 inflammasome complex (Fig. 5A to C). The activated NLRP3 inflammasome mediates the inflammatory response via downstream IL-1β, IL-18, and GSDMD. To examine the effect of THz photons on the execution of inflammatory responses via the NLR signaling pathway, we quantified the levels of its downstream effector components using enzyme-linked immunosorbent assay (ELISA). Consistent with the suppression of the inflammasome, THz photons substantially down-regulated the expression of pro-inflammatory cytokine precursors pro-IL-1β and pro-IL-18, as well as their mature forms, IL-1β and IL-18 (Fig. 5D). Similarly, THz photons decreased GSDMD expression by 21% compared to TBI controls (Fig. 5E). Taken together, these results demonstrate that THz photons effectively inhibit the NLRP3 signaling pathway and its downstream inflammatory outputs. Furthermore, we also found that THz photons increased the cAMP level by approximately 47.2%, compared with TBI mice (Fig. 5F). In addition, at 3 and 14 dpi, the TBI+THz group exhibited elevated cAMP levels and decreased expression of inflammasome-related molecules relative to the TBI group (Figs. S6 and S7). The binding of cAMP to the NLRP3 NOD-binding domain directly inhibits inflammasome assembly [29]. cAMP can also promote the ubiquitination and degradation of NLRP3 [30]. Moreover, NLRP3 is phosphorylated by cAMP through its principal effector protein kinase A, which attenuates the ATPase function of NLRP3 [31]. In conclusion, increased cAMP levels can mediate the inhibition of the NLRP3 inflammasome (Fig. 5G). Collectively, these results indicate that THz photons can regulate the cAMP/NLRP3 signaling pathway.

**Fig. 5.**
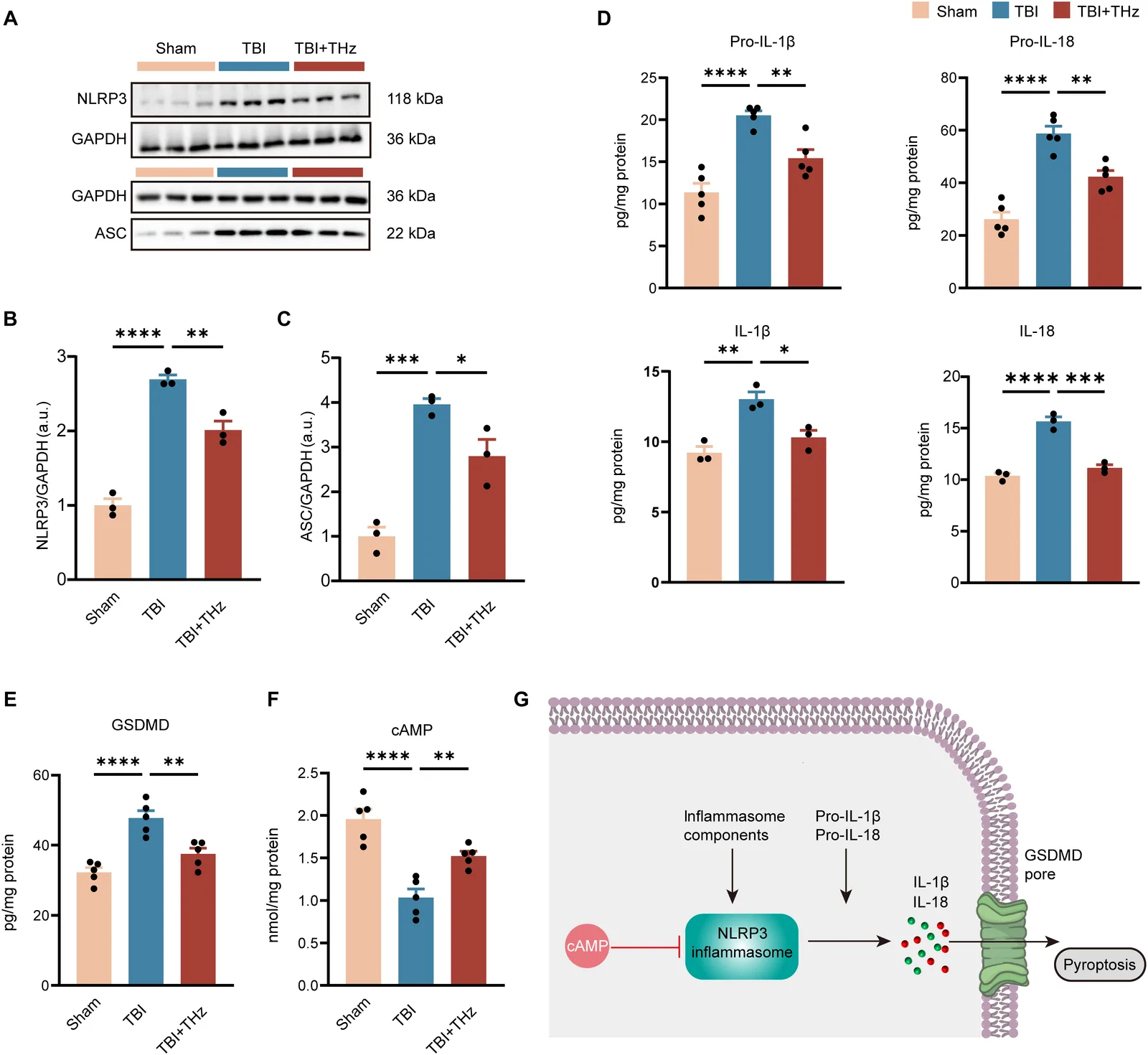
Terahertz (THz) photons inhibit NLR and pyrin domain-containing protein 3 (NLRP3) inflammasome activation by promoting cyclic adenosine monophosphate (cAMP) production. (A) The western blot of NLRP3 and apoptosis-associated speck-like protein containing a CARD (ASC). (B) The relative protein expression of NLRP3 (*n* = 3). (C) The relative protein expression of ASC (*n* = 3). (D) The protein concentrations of pro-interleukin-1β (pro-IL-1β; *n* = 5), pro-interleukin-18 (pro-IL-18; *n* = 5), IL-1β (*n* = 3), and IL-18 (*n* = 3). (E) The protein concentration of gasdermin D (GSDMD; *n* = 5). (F) The concentration of cAMP (*n* = 5). (G) Schematic diagram of the cAMP–NLRP3 signaling pathway. One-way analysis of variance (ANOVA) with Bonferroni post hoc test. Data are expressed as mean ± standard error of the mean (SEM).*P < 0.05; **P < 0.01; ***P < 0.001; ****P < 0.0001.

### THz photons modulate AC9 of microglia

TBI leads to microglial accumulation in the injured lesion [25]. To verify whether THz photons regulate the cAMP signaling pathway in microglia, we irradiated activated BV2 cells by THz photons in vitro. First, BV2 cells was activated by lipopolysaccharide (LPS) and adenosine triphosphate (ATP). ELISA revealed that the treatment of LPS + ATP markedly decreased the level of cAMP (Fig. 6A) and increased the concentration of NLRP3 (Fig. 6B) and the downstream effector molecules (GSDMD, IL-1β, and IL-18) respectively (Fig. 6C to E). In contrast, THz photons markedly elevated the cAMP concentration while reducing the expression levels of these inflammasome-associated proteins in LPS + ATP-treated BV2 cells (Fig. 6A to E). Adenylyl cyclase (AC) is the direct upstream molecule of cAMP and catalyzes the conversion of ATP to cAMP, thereby initiating the cAMP signaling pathway [13]. BV2 cells transfected with siAC9 to down-regulate AC9 expression (Fig. S8), and then exposed to THz photons, exhibited reduced cAMP levels and increased NLRP3 inflammasome-associated proteins compared to LPS + ATP- and THz-photon-treated BV2 cells (Fig. 6A to E). These findings indicate that THz may predominantly modulate AC9 (a major AC isoform in microglia) function in microglia, which further mediates the cAMP–NLRP3 signaling cascade. To verify whether THz photons directly up-regulate cAMP concentration by regulating AC9, we constructed a plasmid overexpressing AC9 (Fig. 6F) and transfected it into HEK-293T cells. THz photons did not markedly increase the cAMP concentration in HEK-293T cells (Fig. 6G); however, it markedly increased cAMP levels in 293T cells overexpressing AC9 (Fig. 6H). These results indicate that THz photons facilitate cAMP elevation through AC9 in microglia, thereby further inhibiting the inflammasome.

**Fig. 6.**
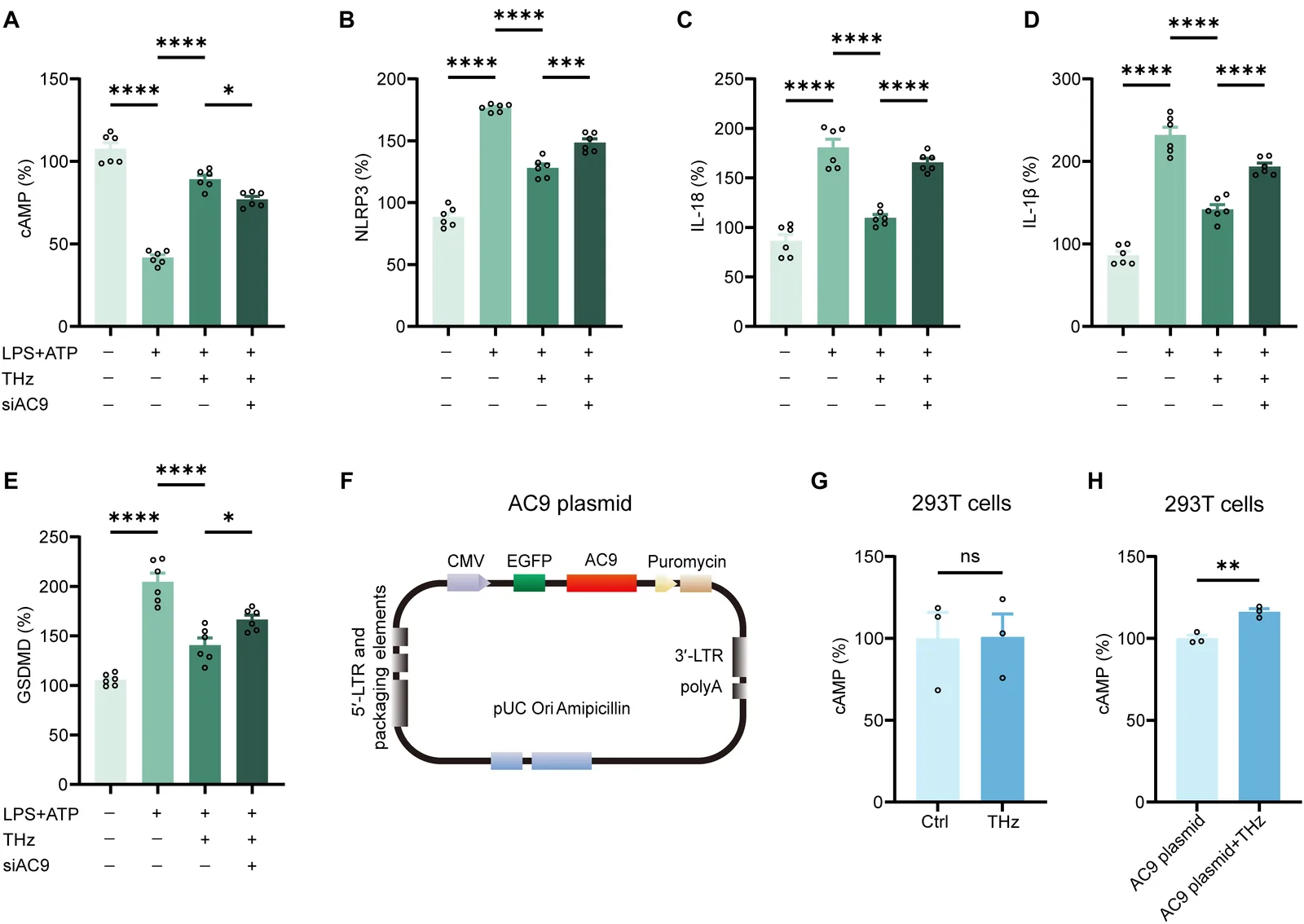
Terahertz (THz) photons modulate adenylyl cyclase type 9 (AC9) of microglia. (A) Cyclic adenosine monophosphate (cAMP) (%) in BV2 microglia (*n* = 6). (B) NLR and pyrin domain-containing protein 3 (NLRP3) (%) in BV2 microglia (*n* = 6). (C) Interleukin-18 (IL-18; %) in BV2 microglia (*n* = 6). (D) Interleukin-1β (IL-1β; %) in BV2 microglia (*n* = 6). (E) Gasdermin D (GSDMD; %) in BV2 microglia (*n* = 6). (F) Schematic of the AC9 plasmid. (G) The relative expression of cAMP in HEK-293T cells and THz-treated HEK-293T cells (*n* = 3). (H) The relative expression of cAMP in AC9-plasmid-transfected HEK-293T cells and THz-treated and AC9-plasmid-transfected HEK-293T cells (*n* = 3). (A) to (E) were analyzed by one-way analysis of variance (ANOVA) with Tukey post hoc test. (G) and (H) were analyzed by a 2-sided, unpaired Student *t* test. Data are expressed as mean ± standard error of the mean (SEM). ns, not significant; *P < 0.05; **P < 0.01; ****P < 0.0001.

### Biosafety evaluation of THz photons

First, the temperature elevation was detected in the irradiated region. The measurement results revealed that the temperature elevation reached approximately 2 °C following 10 min of irradiation (Fig. 7A). Other previous studies have demonstrated that visible-light or THz irradiation can induce a moderate brain temperature increase (~2 °C) [18,20]. A temperature rise of this magnitude neither alters neuronal activity nor generates thermal effects [18,20,32,33]. To further evaluate the biosafety of THz photons, sham mice, which were subjected to craniectomy to expose brain tissue without CCI, were administered THz photons for 3 consecutive days. On the following day after THz, the brains were harvested for biosafety evaluation. Due to intracranial pressure changes and mild brain tissue displacement caused by craniotomy, sham mice exhibited mild cellular damage and inflammatory responses. Hematoxylin and eosin (H&E) staining revealed that sham mice exhibited mild nuclear pyknosis and karyolysis, whereas the Sham+THz group showed alleviation rather than exacerbation of these features (Fig. 7B). Furthermore, compared with sham mice, the number of astrocytes in the Sham+THz group did not show significant difference (Fig. 7C), whereas the number of microglia slightly decreased (Fig. 7D), indicating that THz photons did not induce astrogliosis or microglial proliferation. These data collectively demonstrate that the THz photons employed in this study exhibit good biosafety.

**Fig. 7.**
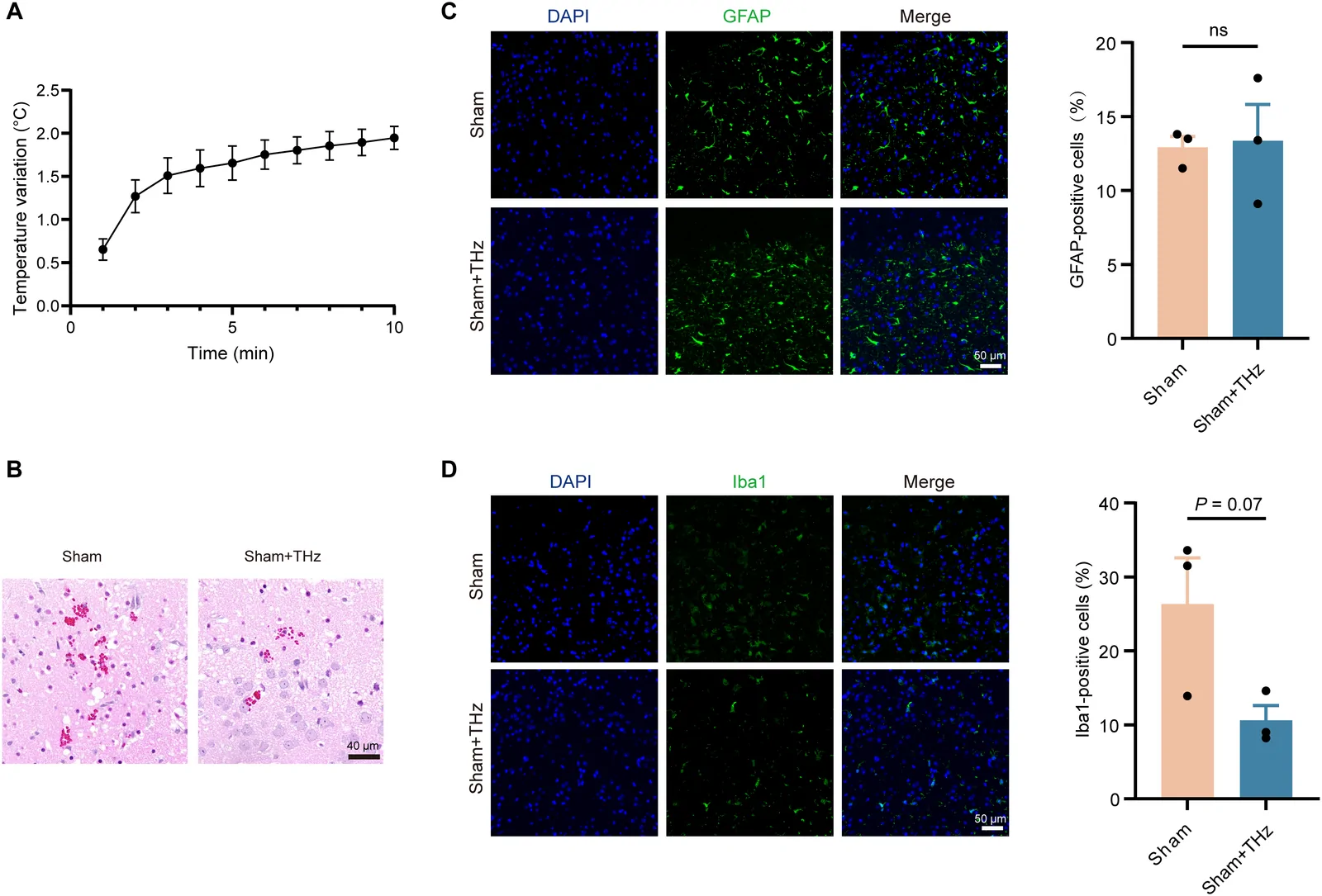
Biosafety evaluation of terahertz (THz) photons. (A) THz-irradiation-induced temperature rise (*n* = 3). (B) Representative hematoxylin and eosin (H&E) images in the Sham and Sham+THz groups. Scale bar = 40 μm. (C) Representative immunofluorescence images and quantitative analysis for glial fibrillary acidic protein (GFAP)-positive cells in the Sham and Sham+THz groups (*n* = 3). Scale bar = 50 μm. (D) Representative immunofluorescence images and quantitative analysis for ionized calcium-binding adapter molecule 1 (Iba1)-positive cells in the Sham and Sham+THz groups (*n* = 3). Scale bar = 50 μm. Data are expressed as mean ± standard error of the mean (SEM). Data were analyzed by a 2-sided, unpaired Student *t* test. ns, not significant.

## Discussion

TBI not only is an acute condition but is also increasingly recognized as a chronic traumatic encephalopathy [34]. In both acute and chronic phases, neuroinflammation serves a critical function and acts as a driver that exacerbates secondary brain injury [35]. In this study, we demonstrate that THz photons alleviate neuroinflammation and facilitate the activation of anti-inflammatory M2 microglia, thereby mitigating secondary injury and improving cognitive outcomes. This effect of THz photons involves the modulation of AC9 and the promotion of cAMP production, which subsequently inhibits NLRP3 inflammasome activation (Fig. 8).

**Fig. 8.**
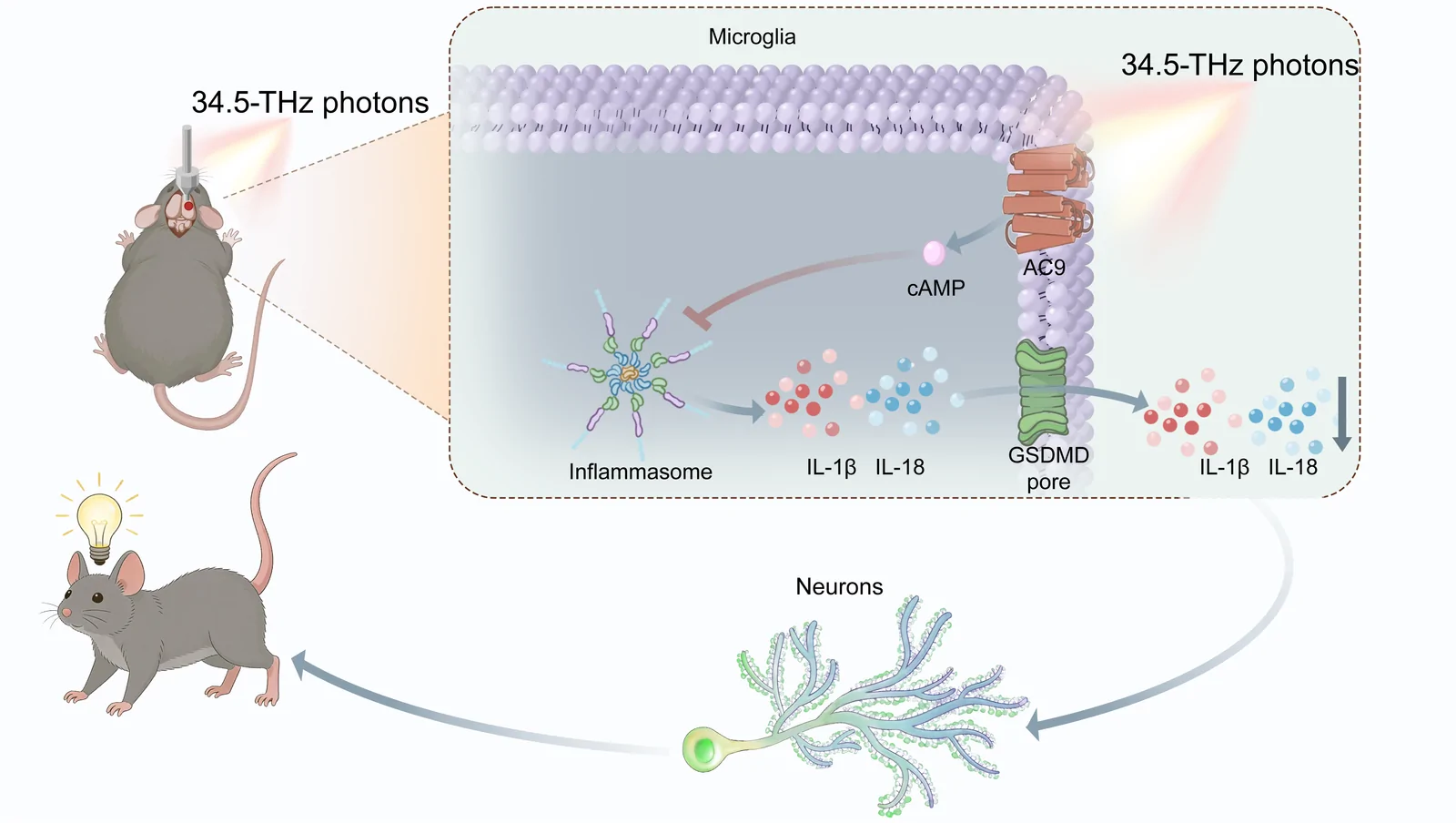
Schematic illustration of the mechanism by which 34.5-THz photons ameliorate traumatic brain injury (TBI). Terahertz (THz) photons modulate adenylyl cyclase type 9 (AC9) in microglia and promote the production of cyclic adenosine monophosphate (cAMP), which inhibits inflammasome activation and decreases downstream inflammatory effector components, ultimately alleviating pathological and cognitive impairments in TBI mice.

The relationship between AC and NLRP3 is inherently complex. Both transmembrane and soluble forms of AC catalyze the cyclization of ATP to cAMP. Accumulating evidence suggests that AC-derived cAMP directly interacts with NLRP3 and impedes inflammasome formation. Down-regulation of intracellular cAMP eliminates such repression, thereby identifying cAMP as an essential modulator of NLRP3 inflammasome activity [29]. Another study demonstrated that forskolin, an AC activator, elevates intracellular cAMP levels and suppresses NLRP3 inflammasome activation in a dose-dependent manner. This effect was effectively abrogated by the AC inhibitor KH7 [30]. Another study reported that cAMP produced by AC mediates the inhibition of NLRP3 inflammasome activation through its primary downstream effector protein kinase A [31]. Collectively, these findings establish cAMP as a negative regulator of the NLRP3 inflammasome. Our results further postulate that THz photons involve the AC9/cAMP/NLRP3 signaling axis in microglia. Specifically, THz photons elevate intracellular cAMP levels and suppress NLRP3 inflammasome activation. Critically, this NLRP3 inflammasome inhibitory effect, as well as the THz-induced cAMP elevation, was markedly attenuated upon siAC9 (Fig. 6A to E). We also found that THz photons increased the cAMP level in HEK-293T cells transfected with the AC9 plasmid (Fig. 6H). These findings substantiate that AC9 is involved in the biological effects of THz irradiation. It is noteworthy that the present work does not provide direct evidence for the in vivo molecular interaction between THz and AC9. Given the rapid advancement of microscopy technologies, we envision that future studies will be able to directly visualize such conformational dynamics in biomolecules upon THz exposure.

Using RNA sequencing and ELISAs, we found that the cAMP signaling pathway was markedly up-regulated following THz photons (Figs. 4C and 5G). The cAMP signaling pathway plays an important role in the central nervous system. Studies have demonstrated that the cAMP pathway in neurons regulates neuroplasticity, neuronal survival, myelination, energy expenditure, neurotransmission, and glucose homeostasis while those in astrocytes and microglia perform a critical function in neuroprotection, energy supply, and immune response [36–39]. Noteworthily, regulation of cAMP has emerged as a promising therapy for modulating inflammatory diseases [40,41]. A previous study demonstrated that the level of cAMP decreases following TBI and the prevention of cAMP hydrolysis improves cognitive outcomes [42]. In our study, THz increased the level of cAMP, therefore markedly inhibiting NLRP3 inflammasome activation (Figs. 5 and 6). NLRP3 exhibited marked changes at the transcriptomic, proteomic, cellular, and in vivo levels, suggesting that it may serve as a core mediator of the neuroprotective effects. However, it should be noted that cAMP is a key second messenger that regulates a lot of downstream pathways [43–45] and may exert neuroprotection through multiple mechanisms, such as promoting neurite outgrowth via the cAMP signaling pathway [13]. This suggests that cAMP may exert neuroprotective effects through other pathways as well.

Inflammasomes act as molecular scaffolds that mediate the activation of pro-inflammatory molecules [9]. Multiple studies have displayed that inflammasome signaling pathways are strongly activated in TBI models [46–49]. Furthermore, TBI-mediated inflammasome complex activation can result in neurodegeneration and tau phosphorylation, which carries longer-term implications for TBI survivors [50]. Therefore, the inhibition of the NLRP3 inflammasome contributes to preventing the development of chronic neuroinflammation. Through RNA sequencing and proteomic analyses, we found that THz photons mainly regulate inflammasome signaling pathway (Fig. 4). Western blot and ELISA analyses further validated that THz photons markedly down-regulated the expression of inflammasome-associated proteins (including NLRP3 and ASC) in TBI mice, along with a marked reduction in the levels of downstream inflammasome mediators (IL-1β, IL-18, and GSDMD) (Fig. 5). These results indicate that THz photons inhibit the activation of inflammasome signals in TBI mice. To further validate these in vivo observations, we treated activated BV2 microglial cells with THz photons. Our data revealed that THz photons similarly down-regulated the expression levels of inflammasome-related proteins in BV2 cells (Fig. 6). Collectively, these results indicate that the inhibition of the inflammasome signaling pathway in microglia by THz photons constitutes a major mechanism by which it mitigates neuroinflammation after TBI. Additionally, although the present study observed the down-regulation of IL-1β and IL-18, pyroptosis-specific evidence is lacking. Moreover, THz irradiation may exert neuroprotective effects through multiple mechanisms, including the inhibition of pyroptosis, ferroptosis, and apoptosis. Future studies using both in vitro and in vivo models will be conducted to further verify the regulatory effect of THz on pyroptosis.

Neuroinflammation is mainly caused by resident cells in the brain and exogenous infiltrating cells. Activated microglia gather at the injury site, and a pro-inflammatory state develops over time, leading to long-term functional deficits and neurodegeneration [35]. Our results showed that TBI mice irradiated with THz photons exhibited a marked reduction of Iba1^+^ cells at 7 dpi (Fig. 3A). Through RT-qPCR and IF staining, we found that THz photons promoted anti-inflammatory M2 activation (Fig. 3C to G). Accumulating evidence indicates that the activity of M2-like responses declines over time following brain injury, whereas pathological M1-like effects can persist chronically [51,52]. Our results indicate that THz photons can reverse this trend, regulate the polarization dynamics of microglia, and promote tissue repair by facilitating the activation of M2-like effects. Notably, the phenotype of microglia following TBI is highly complex [53]. The M1/M2 classification primarily describes functional biases toward pro- or anti-inflammatory responses. Future studies should therefore employ flow cytometry or single-cell RNA sequencing to characterize disease-associated microglia (DAM). DAM may coexpress both M1-like and M2-like signature genes and may exhibit distinct features under TBI conditions compared with those observed in neurodegenerative diseases [54]. For instance, a recent study has identified a novel C5aR1^+^ DAM-like subset that promotes neuroinflammation in acute brain injury [55]. Therefore, the regulatory effect of THz photons on a more precise classification of microglial phenotypes rather than the simple binary classification merits further investigation. Moreover, infiltrating immune cells participate in neuroinflammation through various mechanisms [56]. We further analyzed the dynamic changes in the number of infiltrating cells by flow cytometry. The results demonstrated that THz photons reduced the levels of the key chemokine CXCL1, concomitant with a reduction in the number of neutrophils at the injury site, albeit without statistical significance (Fig. 3H to J and Fig. S3). THz treatment did not markedly reduce the numbers of other infiltrating immune cells, including macrophages, CD4^+^ T cells, CD8^+^ T cells, and Tregs (Fig. S4). These findings suggest that THz alleviates neuroinflammation mainly by regulating microglia, not infiltrating immune cells.

The beneficial effects of THz irradiation in TBI may also involve other regulatory mechanisms. THz irradiation may promote the growth of neurons and reduce neuronal damage [13]. THz irradiation may also modulate another major type of resident cell, namely, astrocytes. Astrocytes exist in 2 distinct phenotypic states: neurotoxic astrocytes and neuroprotective astrocytes. Specifically, neuroprotective astrocytes can suppress microglial activation and the differentiation of downstream neurotoxic astrocytes [39]. Activated microglia exert phagocytic activity to prune astrocyte processes, impairing the functional integrity of glutamate transporter 1. This compromise induces excessive glutamate accumulation in the synaptic cleft [57], which may trigger glutamate excitotoxicity and subsequent neuronal damage. Microglia and astrocytes have complex interactions. THz irradiation may exert multiple regulatory effects by inhibiting the interaction between microglia and astrocytes while directly acting on neurons. In the future, we plan to study other potential regulatory mechanisms and spatiotemporal characteristics of THz irradiation.

As an emerging physical therapy modality, THz radiation has yet to be investigated for its effects on mechanosensitive ion channels. Mechanosensitive ion channels are integral membrane proteins expressed on living cells that transduce mechanical stimuli into biochemical signals, thereby regulating a wide range of cellular activities [58]. For instance, Piezo1, a well-characterized mechanosensitive channel, is susceptible to various physical cues, including ultrasound, electrical fields, ionizing radiation, and magnetic energy, and can be induced to transition from a closed to an open state. Upon activation, the Piezo1 channel permits the flux of Ca^2+^, K^+^, and Na^+^ across the plasma membrane [59]. Notably, Piezo1 has been implicated in mediating neuroinflammation [59]. Both amyloid-β and LPS up-regulate Piezo1 expression [60]. It is plausible that Piezo1 coordinates Toll-like receptor signaling and facilitates Ca^2+^ influx, which in turn may modulate cellular immune activity [60]. Elevated cytoplasmic Ca^2+^ levels resulting from Piezo1 activation subsequently trigger the nuclear factor-κB pathway, leading to increased expression of NLRP3, pro-IL-1β, and pro-IL-18. Moreover, Ca^2+^ promotes inflammasome assembly, enhances caspase-1 activation, and augments the secretion of IL-1β [61]. Conversely, inhibition of Piezo1 may be responsible for the suppression of NLRP3 signaling. Given that THz radiation is a nonionizing physical stimulus, whether it exerts any influence on mechanosensitive ion channels constitutes a critical avenue for future research. Elucidating this mechanism would substantially advance our understanding of the upstream biosensory processes underlying THz-based therapeutic applications.

Notably, the penetration depth of THz radiation into biological tissues is intrinsically limited [18,20], which poses a considerable challenge for delivering THz to deep-brain structures. Although fiber optics or other invasive approaches may be used to target specific regions directly, the applicability of THz remains constrained under certain conditions—for example, in diffuse brain injury, where focal delivery is not feasible. Therefore, the clinical indications for THz application must be carefully evaluated. Moreover, given that the human brain is substantially larger than the mouse brain, ensuring that THz energy reaches a sufficiently broad brain region with adequate fluence represents a major hurdle for clinical translation. In addition, because THz is directly applied to living tissue, in vivo tissue dosimetry is extremely challenging, making the actual energy deposition difficult to determine. Overcoming these obstacles will likely require the development of high-power quantum cascade lasers (QCLs) as well as more efficient transmission and delivery methodologies.

In summary, our research identifies THz photons as a promising therapeutic approach for TBI. THz photons induce an increase in cAMP level in a manner associated with AC9, which inhibits the activation of the NLRP3 inflammasome and prevents the inflammatory cascade, thereby promoting functional recovery in the TBI mice. Our research provides a nonpharmacological and noninvasive therapy that inhibits the NLRP3 inflammasome. This method has potential in the treatment of TBI and other forms of acute bran injury.

## Methods

### Animals

Wild-type C57BL/6 mice, aged 8 to 10 weeks, were obtained from SPF (Beijing) Biotechnology Co., Ltd and maintained under a 12-h light/12-h dark cycle. All mice had free access to adequate water and food. All animal care and experimental procedures were conducted in strict accordance with the institutional guidelines and were approved by the Institutional Animal Care and Use Committee under license IACUC-DWZX-2025-581.

### Controlled cortical impact

CCI was performed as reported previously [62]. Briefly, mice were anesthetized with 1.25% tribromoethanol solution (0.2 ml/10 g) intraperitoneally and remained stable. A longitudinal incision was made in the scalp, and the skull was exposed. A 3-mm-diameter craniotomy was performed in the right hemisphere using an electromagnetic drill, and the bone flap was carefully elevated. CCI was then induced at a site 2 mm caudal to bregma and 2 mm lateral to the right of sagittal suture. The impact was delivered using a pneumatic CCI device (RWD Life Science Co., Ltd.) with a 2-mm-diameter tip, at a velocity of 4.5 m/s, a deformation depth of 2.0 mm, and a dwell time of 500 ms.

### Magnetic resonance imaging

MRI was performed on mice from the Sham and TBI groups at 3 d post-CCI. Briefly, mice were gently restrained and placed in an anesthesia induction box and pre-anesthetized with a mixed gas of 2.5% isoflurane and oxygen. Mice were fixed in a prone position on the animal bed, and anesthesia was maintained with a mixed gas of 1.5% to 2.0% isoflurane and oxygen. A physiological status monitoring system (PC-SAM32 software) was employed for the real-time assessment of respiratory rate in mice. The respiratory rate was controlled between 60 and 80 breaths per min. Multimodal MRI was performed on the mouse brain using PharmaScan 70/16 US (Bruker, Germany). The T2 structural scan sequence is T2_TurboRARE, with the following scanning parameters: repetition time/echo time, 3,353/35 ms; slice thickness, 0.5 mm; field of view, 22 × 22 mm; image size, 256 × 256; and averages, 4.

### THz photon exposure in vivo

The THz photon system consisted of a QCL source and an optical delivery assembly. The QCL was operated at 34.5 THz with a pulse duration of 2 μs, a repetition rate of 200 kHz, and a duty cycle of 40% [20]. The THz photons emitted by the QCL was reflected by multiple gold mirrors and finally focused to a 3.0-mm-diameter beam through a convex lens. The beam was delivered vertically to the injury site at a power density of 4 or 7 mW/mm^2^. THz photon exposure was performed at 1, 24, and 48 h postinjury, with each session lasting 10 min.

### Morris water maze

The MWM was used to evaluate spatial learning and reference memory, which includes a 5-d acquisition phase followed by a probe trail [3,63]. During the 5-d acquisition phase, mice underwent 4 trials per day from 4 different start locations, with a maximum of 60 s to locate the underwater platform. Mice that failed to find the platform within the time limit were gently guided to the platform and allowed to remain for 15 s; 24 h after the last acquisition day, a probe trial was conducted with the platform removed. Mice were reintroduced to the maze from a novel start location and allowed to explore freely for 60 s. Exclusion criteria were defined as physical weakness or inability to perform the swimming task effectively. Video recordings were captured throughout the experiment. Movement trajectories were tracked and analyzed using the Smart analysis software (v3.0). The following parameters were recorded: latency to target, target crossings, and the percent of time or distance in the target quadrant.

### NOL test

The NOL and MWM tests were performed on separate cohorts of mice. The NOL test was used to assess spatial memory. First, each mouse was placed in the experimental arena for a 5-min adaptation. Next, 2 identical objects were placed in the arena, and the mouse was allowed to familiarize itself with these objects for 5 min. After a 1-h retention interval, 1 of the 2 previously familiar objects was moved to a novel location, and the mouse was returned to the arena for the exploration trial, which was terminated once the cumulative exploration duration for the 2 objects reached 20 s. Throughout the experiment, mice’s behaviors were recorded using a camera, and the collected data were analyzed with the Smart analysis software (v3.0). The location recognition index was calculated as the ratio of exploration time for the NOL to the total exploration time for both object location.

### IF staining and TUNEL staining

Following deep anesthesia by 1.25% tribromoethanol solution, mice were transcardially perfused with phosphate-buffered saline (PBS) and 4% paraformaldehyde in sequence. Brains were collected and fixed in 4% paraformaldehyde for 24 h and then dehydrated sequentially in 20% and 30% sucrose solutions. After dehydration, brains were frozen in optimal cutting temperature compound (Sakura, 4583) and stored at −80 °C. For IF staining, brains were sliced into 40-μm sections using a cryostat microtome (Leica Biosystems, CM1950). Then, sections were blocked in with a 5% bovine serum albumin solution, containing 0.5% Triton X-100, and were incubated overnight at 4 °C with primary antibodies in blocking buffer. Sections were washed 3 times with PBS and incubated with secondary antibody solution for 2 h at room temperature in a humid box. Following incubation with secondary antibodies, brain sections were washed 3 times with PBS. After washing, sections were mounted on microscope slides, and coverslips were mounted with an antifading solution containing 4′,6-diamidino-2-phenylindole (DAPI) (Beyotime, P0131). Sections were also stained with TUNEL (Beyotime, C1088) according to its kit protocol. Images were observed and captured using a Pannoramic MIDI slide scanner (3DHISTECH Ltd, Budapest, Hungary). The primary and secondary antibodies used were as follows: Iba1 (1:1,000, Abcam, ab283319), CD16 (1:500, Abcam, ab223200), Arg1 (1:500, Proteintech, 16001-1-AP), glial fibrillary acidic protein (1:1,000, Proteintech, 16825-1-AP), Cy3 goat anti-rabbit immunoglobulin G (IgG) (1:200, Invitrogen, A10520), and fluorescein isothiocyanate goat anti-mouse IgG (1:200, Invitrogen, F2761).

### Nissl staining

To quantify the brain damage volume at 21 dpi, 50-μm coronal sections were prepared at 200-μm intervals to cover the entire injured tissue. Sections were stained with Nissl (Beyotime, C0117). After Nissl staining, the sections were imaged. The areas of the ipsilateral and contralateral hemispheres were measured by the ImageJ software for each section. The lesion volume was calculated via numerical integration of the lesion areas (the area of the contralateral hemisphere minus the area of the ipsilateral hemisphere) across consecutive sections.

### Golgi staining

Brains were obtained and immediately fixed in Golgi fixative for 24 h. Subsequently, the brains were cut into 2- to 3-mm-thick tissue blocks, which were then immersed in Golgi staining solution (Macklin, R669626) for 14 d with the solution replaced every 3 d. Next, the tissue blocks were treated with tissue processing solution at 4 °C in the dark for 3 d. Finally, 60-μm-thick sections were cut for development by Golgi developer solution. Stained sections were visualized using a Pannoramic MIDI slide scanner. Spine density and Sholl analysis were conducted following established methods [13]. For dendritic spine density analysis, 3 morphologically intact neurons with well-defined dendritic segments were selected from the peri-lesion area per mouse. The length of each dendritic segment and the number of spines along it were measured, and spine density was calculated as the number of spines per unit dendritic length. For Sholl analysis, 3 morphologically intact neurons were similarly chosen from the peri-lesion area in each animal. A series of 10 concentric circles with an interradius interval of 10 μm was centered on the soma, and the number of dendritic intersections with each circle was recorded. The total number of intersections across all 10 circles was then summed for each neuron. In sham mice, the same corresponding regions were analyzed as in the TBI group.

### H&E staining

H&E staining was conducted as described in the manufacturer’s kit protocol (Beyotime, C0105S). Briefly, paraffin-embedded brain sections (5 μm) were deparaffinized, rehydrated, and stained with hematoxylin for 5 min, followed by eosin for 2 min. After dehydration and clearing, sections were mounted with neutral resin. Histological examination was performed under a light microscope.

### Western blotting

The lesion area of brain tissue was collected and rapidly frozen in liquid nitrogen and then stored at −80 °C. Protein was extracted by homogenization in ice-cold radioimmunoprecipitation assay buffer containing protease inhibitor (Roche, 04693124001). The resulting lysates were centrifuged at 13,000 rpm and 4 °C for 15 min, and the supernatants containing soluble proteins were collected. Equal amounts of protein (20 μg per sample) were separated via sodium dodecyl sulfate–polyacrylamide gel electrophoresis and then electrotransferred onto 0.45 μm polyvinylidene fluoride membranes (Merck Millipore, IPVH00010). After blocking with 5% nonfat milk in tris-buffered saline containing 0.1% Tween-20 (TBST) for 1 h at room temperature, the membranes were incubated overnight at 4 °C with specific primary antibodies. Subsequently, the membranes were rinsed 3 times with TBST and incubated with the secondary antibody for 1 h at room temperature. Protein bands were visualized using a chemiluminescence imaging system (Tanon, 5200) and quantified by using ImageJ.

The primary antibodies were glyceraldehyde-3-phosphate dehydrogenase (GAPDH) antibody (1:5,000, Proteintech, 10494-1-AP), NLRP3 antibody (1:1,000, Abcam, ab263899), ASC antibody (1:1,000, Abcam, ab309497), and AC9 antibody (1:1,000, Abcam, ab191423). The secondary antibody is horseradish peroxidase-linked anti-rabbit IgG (1:5,000, Cell signaling technology, 7074S).

### Enzyme-linked immunosorbent assay

Protein concentrations were quantified using a bicinchoninic acid protein assay kit (MEIMIAN, MM-9227B). ELISA was performed by commercially available ELISA kits (MEIMIAN, Jiangsu, China) to measure the following proteins and molecules: NLRP3, ASC, pro-IL-1β, pro-IL-18, IL-1β, IL-18, cAMP, GSDMD, and CXCL1.

### Reverse transcription quantitative PCR

RNA was extracted directly from bulk peri-lesional brain tissue. Total RNA was extracted using the RNA Extraction Kit (Servicebio, G3689), and complementary DNA (cDNA) was synthesized using All-in-one 1st Strand cDNA Synthesis SuperMix (Novoprotein, E047) according to the protocol of the kit. RT-qPCR was performed using the SYBR qPCR SuperMix (Novoprotein, E096-01B) on an FMR3 Real-Time quantitative thermal cycler (Suzhou Molarray). Gene expression levels were quantified using the 2^−∆∆CT^ method, with GAPDH mRNA serving as the internal control gene. The primers used are listed below:

• CD16 forward: 5′-TTTGGACACCCAGATGTTTCAG-3′
• CD16 reverse: 5′-GTCTTCCTTGAGCACCTGGATC-3′
• CD206 forward: 5′-CAAGGAAGGTTGGCATTTGT-3′
• CD206 reverse: 5′-CCTTTCAGTCCTTTGCAAGC-3′
• AC9 forward: 5′-GGTGGTGGGACCCCAAATTC-3′
• AC9 reverse: 5′-CCAAACCTGAAACGGAAGCGG-3′
• GAPDH forward: 5′-AGGTCGGTGTGAACGGATTTG-3′
• GAPDH reverse: 5′-TGTAGACCATGTAGTTGAGGTCA-3′

### Flow cytometry

The injured brain tissue was isolated, minced, and dissociated in 2.5 ml of 1% Dulbecco’s PBS supplemented with 5 μl of collagenase IV and 15 μl of DNase, followed by incubation with shaking for 30 min at room temperature. After dissociation, the cells were filtered through a 40-μm cell strainer, centrifuged at 400 × *g* for 5 min to remove the supernatant, and subsequently separated using 24% Percoll. For flow cytometry, cells were stained with the following antibody panel: BB515 anti-mouse CD45 (BD, 564590), BV605 anti-mouse CD3 (BD, 564009), BUV496 anti-mouse CD4 (Invitrogen, 364-0042-82), BV510 anti-mouse CD8 (BioLegend, 100752), PE anti-mouse FoxP3 (BioLegend, 126404), BV785 anti-mouse CD25 (BioLegend, 102051), RB705 anti-mouse F4/80 (BD, 570288), BV421 anti-mouse CD11b (BioLegend, 101236), and BV711 anti-mouse Ly-6G (BioLegend, 127643). Flow cytometry was performed on an Aurora CS full spectrum cell sorting system (Cytek).

### RNA sequencing

Total RNA was extracted from lesion or brain tissues (*n* = 3 per group) at 7 dpi. RNA integrity was assessed using Agilent 2100 Bioanalyzer (Agilent Technologies). The cDNA libraries were constructed following a previous protocol [64], including mRNA enrichment via oligo(dT) beads, fragmentation, reverse transcription, end repair, add A tail and adaptor ligation, and PCR amplification. Libraries were first quantified using Qubit 2.0 Fluorometer. Subsequently, Agilent 2100 Bioanalyzer was used to detect the insert size of the libraries. Once the insert size met the expected criteria, RT-qPCR was employed for accurate quantification of the effective concentration of the libraries to ensure library quality.

Different libraries were pooled according to their effective concentrations, followed by Illumina sequencing based on sequencing by synthesis. Differentially expressed genes between groups were identified with thresholds of |log_2_(fold change)| ≥ 0 and false discovery rate (FDR)-adjusted *P* value < 0.05. GO and KEGG enrichment analyses were performed to analyze the biological functions and potential signaling pathways of target genes.

### Proteomic analysis

Sample preparation, protein extraction, protein digestion, and peptide desalting were performed as previously reported [65,66]. Data-independent acquisition (DIA) analysis was performed on a liquid chromatography–tandem mass spectrometry platform [26]. DIA raw data were processed with the generated spectral library for peptide and protein identification and quantification. Differentially expressed proteins were identified with thresholds of |log_2_(fold change)| ≥ 0.26 and FDR-adjusted *P* value < 0.05. Functional enrichment analysis of differentially expressed proteins was performed, including GO and KEGG pathway annotations.

### Temperature detection

The method for detecting temperature elevation was adapted from previous studies [20]. Briefly, a temperature-sensitive probe was used to monitor the temperature within the irradiated area. The probe was placed beneath the THz exposure zone to prevent direct irradiation of the sensor. Upon turning on the THz source, temperature variations were recorded in real time via the accompanying software.

### Cell culture

BV2 cell lines and HEK-293T cell lines were purchased from Procell. All cell lines were cultured in Dulbecco’s modified Eagle’s medium (Gibco, 10569010) supplemented with 10% fetal bovine serum (Gibco, 16000044) and 1% penicillin–streptomycin (Gibco, 15140122) at 37 °C with 5% CO_2_.

### BV2 cell activation and THz photon exposure in vitro

BV2 cells were seeded into 35-mm culture dishes (81156, Ibidi) at a density of 5 × 10^4^ cells/ml, with 500 μl of cell suspension added to each dish. After 12 h, BV2 cells were activated with 1 μg/ml LPS (Sigma-Aldrich, L2880) and 1 mM ATP (Solarbio, A3260) for 12 h [67]. After activation, the medium was replaced, and THz photon exposure was performed with each session lasting 10 min. Briefly, QCL was operated at 34.5 THz with a pulse duration of 2 μs, a repetition rate of 200 kHz, and a duty cycle of 40%. The THz photons emitted by the QCL was reflected by gold mirrors to irradiate from bottom to top, with the beam passing through the culture dish bottom to illuminate cells. The beam power density through the dish bottom was 7 mW/mm^2^.

### Plasmid transfection and THz photon exposure in vitro

HEK-293T cells were seeded into 35-mm culture dishes at a density of 1 × 10^5^ cells/ml, with 500 μl of cell suspension added to each dish. After 12 h, plasmid transfection was performed using AC9 cDNA (GeneCopoeia, EX-Mm34147-Lv103) and Lipofectamine 3000 reagent (Thermo Fisher, L3000015). Briefly, Lipofectamine 3000 reagent and plasmid were diluted in Opti-MEM medium and incubated for 15 min, followed by mixing in a ratio of 1.5 μl of Lipofectamine 3000:1 μg of plasmid. After incubation for 20 min, the plasmid–lipid complex was added into the cells at a plasmid concentration of 1 μg/ml. After 24 h, the medium was replaced, followed by THz photon exposure as noted previously.

### Small interfering RNA transfection and THz photon exposure in vitro

siAC9 was purchased from Beijing Tsingke Biotech Co., Ltd. Briefly, Lipofectamine 3000 reagent and siAC9 were diluted in Opti-MEM medium and incubated for 15 min, followed by mixing in a ratio of 2.0 μl of Lipofectamine 3000:1.0 μl of siAC9. After incubation for 20 min, the siAC9–lipid complex was added into the cells at a siAC9 concentration of 20 nM. After 24 h, the siAC9-transfected BV2 cells were seeded into 35-mm culture dishes at a density of 5 × 10^4^ cells/ml, with 500 μl of cell suspension added to each dish. After 12 h, BV2 cells were activated and exposed to THz photons in vitro as noted previously.

### Statistical analysis

Continuous data with a normal distribution are presented as mean ± standard error of the mean. Detailed analytical methods and *P* values are presented in Table S5. Statistical significance was set at *P* < 0.05. Prior to selecting parametric statistical tests, we examined the normality of data distribution and the homogeneity of variances. Normality was assessed using the Shapiro–Wilk test, and homogeneity of variances was evaluated with Brown–Forsythe test or Bartlett’s test. Data conforming to both assumptions were evaluated using either analysis of variance (ANOVA) (for multiple groups) or the Student *t* test (for 2 groups). For nonnormally distributed data, nonparametric rank-sum tests were applied. When the assumption of variance homogeneity was violated, the Brown–Forsythe and Welch ANOVA (for multiple groups) or Welch’s *t* test (for 2 groups) were used to ensure valid inferences. All statistical analyses were performed using the GraphPad Prism 10 software (GraphPad Software Inc., San Diego, CA, USA).

## Data Availability

The data that support the findings of this study are available from the corresponding authors on reasonable request.
