# Supplementary material for: Terahertz Photons Ameliorate Traumatic Brain Injury and Reduce Neuroinflammation via the Cyclic Adenosine Monophosphate Signaling Pathway
Source: Research (Wash D C). 2026 Oct 27;9:1468. doi: 10.34133/research.1468 (PMC13710765; doi:10.34133/research.1468)
Supplement: Supplementary file 1 [file research.1468.f1.zip › Revised Supplementary Material.docx]

Supplementary Materials for

**Terahertz Photons Ameliorate Traumatic Brain Injury and Reduce Neuroinflammation via cAMP signaling pathway**

**This file includes:**

Figs. S1 to S8

Table S1 to S5

Supplementary Methods

Fig. S1. THz photons did not affect motor function.

(A) Distance and mean speed of MWM. Data were analyzed by one-way ANOVA followed by Bonferroni post-hoc test. Sham (n = 13), TBI (n = 16), TBI+ 4 mW/mm^2^ (n = 15), TBI+ 7 mW/mm^2^ (n = 14). (B) Open field test. Data were analyzed by one-way ANOVA followed by Bonferroni post-hoc test. Sham (n = 6), TBI (n = 11), TBI+ 7 mW/mm^2^ (n = 11). Data are shown as mean ± SEM. ns: not significant.

Fig. S2. THz photons did not affect cerebral blood flow at the injury site.

(A) Blood flow images for TBI and TBI+THz mice. For each group, the left images are the gray images, and the right one is the flux images. Area inside the circle: lesion region. (B) Results of blood flow at different time points. (n = 5). (B) were analyzed by two-sided, unpaired Student’s t-test. Data are shown as mean ± SEM. ns: not significant.

Fig. S3. Flow cytometry scatter plots and quantitative analysis of neutrophils at 3 dpi and 14 dpi.

(A) Flow cytometry scatter plot of neutrophils isolated from the lesion area at 3 dpi and 14 dpi. (B) Quantitative analysis of neutrophils proportion of Sham, TBI and TBI+THz group at 3 dpi. (n=6). (C) Quantitative analysis of neutrophils proportion of Sham, TBI and TBI+THz group at 14 dpi. (n=6). Data are expressed as the mean ± SEM. ns: not significant, *P < 0.05, ***P < 0.001, (one-way ANOVA followed by Bonferroni post-hoc test).

Fig. S4. Flow cytometry scatter plots and quantitative analysis of macrophages, CD8^+^T cells, CD4^+^T cells and Tregs.

(A) Scatter plots of macrophages. (B) Quantitative analysis of macrophages proportion. Sham group *(n=4)*, TBI and TBI+THz group *(n=6)*. (C) Scatter plots of CD8^+^T cells and CD4^+^T cells. (D) Quantitative analysis of CD8^+^T cells proportion. *(n=3)*. (E) Quantitative analysis of CD4^+^T cells proportion. *(n=3)*. (F) Scatter plots of Tregs. (G) Quantitative analysis of Tregs proportion. *(n=3)*. One-way ANOVA with Bonferroni post-hoc test was used. Data are shown as mean ± SEM. ns: not significant, **P < 0.05, *** P < 0.001*.

Fig. S5. The volcano maps of RNA-seq and proteomics.

(A) Volcano map of RNA sequencing. (B) Volcano map of proteomics.

Fig. S6. THz photons regulate cAMP/NLRP3 signaling pathway at 3 dpi.

(A) cAMP (%). (B) NLRP3 (%). (C) ASC (%). (D) GSDMD (%). (E) Pro-IL-18 (%). (F) Pro-IL-1β (%). (G) IL-18 (%). (H) IL-1β (%). All data were expressed as the ratio relative to the Sham group. (mean ± SEM). (n=4). *P < 0.05, **P < 0.01, ***P < 0.001 (two-sided, unpaired Student’s t-test).

Fig. S7. THz photons regulate cAMP/NLRP3 signaling pathway at 14 dpi.

(A) cAMP (%). (B) NLRP3 (%). (C) ASC (%). (D) GSDMD (%). (E) Pro-IL-18 (%). (F) Pro-IL-1β (%). (G) IL-18 (%). (H) IL-1β (%). All data were expressed as the ratio relative to the Sham group. (mean ± SEM). (n=4). *P < 0.05, **P < 0.01, ***P < 0.001 (two-sided, unpaired Student’s t-test).

Fig. S8. siAC9 suppressed AC9 expression at both the mRNA and protein levels.

(A) The relative AC9 mRNA expression. (n=4). (two-sided, unpaired Student’s t-test).

(B) The relative AC9 protein expression. (n=4). (two-sided, unpaired Welch’s t-test). Data are shown as mean ± SEM. **P < 0.01, ***P < 0.001

Table S1. Quantification of Iba1⁺ and Arg1⁺ cells

| groups | Iba1^+^ counts | Iba1^+^ and Arg1^+^ counts | Arg^+^/Iba1^+^ |
| --- | --- | --- | --- |
| TBI | 103 | 8 | 7.8% |
|  | 74 | 9 | 12.2% |
|  | 118 | 7 | 5.9% |
| TBI+THz | 84 | 29 | 34.5% |
|  | 30 | 8 | 26.7% |
|  | 69 | 32 | 46.4% |

Table S2. Quantification of Iba1⁺ and CD16⁺ cells

| groups | Iba1^+^ counts | Iba1^+^ and CD16^+^ counts | CD16^+^/Iba1^+^ |
| --- | --- | --- | --- |
| TBI | 124 | 82 | 66.1% |
|  | 123 | 76 | 61.8% |
|  | 123 | 63 | 51.2% |
| TBI+THz | 68 | 25 | 36.8% |
|  | 55 | 20 | 36.4% |
|  | 56 | 32 | 57.1% |

Table S3. Quantification of GFAP⁺ for biosafety evaluation

| groups | GFAP^+^ counts | DAPI^+^ counts | GFAP^+^/DAPI^+^ |
| --- | --- | --- | --- |
| Sham | 26 | 189 | 13.8% |
|  | 30 | 261 | 11.5% |
|  | 31 | 230 | 13.5% |
| Sham+THz | 33 | 187 | 17.6% |
|  | 28 | 209 | 13.4% |
|  | 23 | 253 | 9.1% |

Table S4. Quantification of Iba1⁺ for biosafety evaluation

| groups | Iba1^+^ counts | DAPI^+^ counts | Iba1^+^/DAPI^+^ |
| --- | --- | --- | --- |
| Sham | 64 | 203 | 31.5% |
|  | 44 | 131 | 33.6% |
|  | 28 | 201 | 13.9% |
| Sham+THz | 15 | 182 | 8.2% |
|  | 21 | 233 | 9.0% |
|  | 25 | 171 | 14.6% |

Table S5. Statistical analyses

| **Figure** | **Analysis** | **Comparison** | **P value** |
| --- | --- | --- | --- |
| Figure 1D  Latency to target | One way ANOVA with Bonferroni *post hoc* analysis | TBI vs Sham | 0.0044 |
|  |  | TBI vs TBI+4mW/mm^2^ | 0.4284 |
|  |  | TBI vs TBI+7mW/mm^2^ | 0.0004 |
| Figure 1D  Target crossing | Kruskal-Wallis test with Dunn post hoc analysis | TBI vs Sham | 0.0092 |
|  |  | TBI vs TBI+4mW/mm^2^ | >0.9999 |
|  |  | TBI vs TBI+7mW/mm^2^ | 0.0532 |
| Figure 1D  Time in target quadrant (%) | One way ANOVA with Bonferroni *post hoc* analysis | TBI vs Sham | 0.0003 |
|  |  | TBI vs TBI+4mW/mm^2^ | 0.4385 |
|  |  | TBI vs TBI+7mW/mm^2^ | 0.0353 |
| Figure 1D  Diatance in target quadrant (%) | One way ANOVA with Bonferroni *post hoc* analysis | TBI vs Sham | 0.0007 |
|  |  | TBI vs TBI+4mW/mm^2^ | 0.2685 |
|  |  | TBI vs TBI+7mW/mm^2^ | 0.0735 |
| Figure 1F | One way ANOVA with Bonferroni *post hoc* analysis | TBI vs Sham | 0.0301 |
|  |  | TBI vs TBI+4mW/mm^2^ | >0.9999 |
|  |  | TBI vs TBI+7mW/mm^2^ | 0.0131 |
| Figure 2B | Unpaired t test | TBI+THz vs TBI | 0.0095 |
| Figure 2C | Unpaired t test | TBI+THz vs TBI | 0.0219 |
| Figure 2D | One way ANOVA with Bonferroni *post hoc* analysis | TBI vs Sham | 0.0003 |
|  |  | TBI vs TBI+THz | 0.0001 |
| Figure 2E | One way ANOVA with Bonferroni *post hoc* analysis | TBI vs Sham | <0.0001 |
|  |  | TBI vs TBI+THz | 0.0137 |
| Figure 3B | One way ANOVA with Bonferroni *post hoc* analysis | TBI vs Sham | <0.0001 |
|  |  | TBI vs TBI+THz | 0.0018 |
| Figure 3C | Brown-Forsythe and Welch ANOVA tests with Dunnett’s T3 *post hoc* analysis | CD16 mRNA  TBI vs Sham | 0.0015 |
|  |  | CD16 mRNA  TBI vs TBI+THz | 0.9942 |
|  | One way ANOVA with Bonferroni *post hoc* analysis | CD206 mRNA  TBI vs Sham | 0.0020 |
|  |  | CD206 mRNA  TBI vs TBI+THz | 0.0257 |
| Figure 3E | Unpaired t test | TBI vs TBI+THz | 0.1164 |
| Figure 3G | Unpaired t test | TBI vs TBI+THz | 0.0107 |
| Figure 3H | One way ANOVA with Bonferroni *post hoc* analysis | TBI vs Sham | <0.0001 |
|  |  | TBI vs TBI+THz | 0.0079 |
| Figure 3J | One way ANOVA with Bonferroni *post hoc* analysis | TBI vs Sham | 0.0182 |
|  |  | TBI vs TBI+THz | 0.1407 |
| Figure 5B | One way ANOVA with Bonferroni *post hoc* analysis | TBI vs Sham | <0.0001 |
|  |  | TBI vs TBI+THz | 0.0040 |
| Figure 5C | One way ANOVA with Bonferroni *post hoc* analysis | TBI vs Sham | 0.0004 |
|  |  | TBI vs TBI+THz | 0.0373 |
| Figure 5D  Pro-IL-1β | One way ANOVA with Bonferroni *post hoc* analysis | TBI vs Sham | <0.0001 |
|  |  | TBI vs TBI+THz | 0.0038 |
| Figure 5D  Pro-IL-18 | One way ANOVA with Bonferroni *post hoc* analysis | TBI vs Sham | <0.0001 |
|  |  | TBI vs TBI+THz | 0.0015 |
| Figure 5D  IL-1β | One way ANOVA with Bonferroni *post hoc* analysis | TBI vs Sham | 0.0203 |
|  |  | TBI vs TBI+THz | 0.0037 |
| Figure 5D  IL-18 | One way ANOVA with Bonferroni *post hoc* analysis | TBI vs Sham | <0.0001 |
|  |  | TBI vs TBI+THz | 0.0002 |
| Figure 5E | One way ANOVA with Bonferroni *post hoc* analysis | TBI vs Sham | <0.0001 |
|  |  | TBI vs TBI+THz | 0.0025 |
| Figure 5F | One way ANOVA with Bonferroni *post hoc* analysis | TBI vs Sham | <0.0001 |
|  |  | TBI vs TBI+THz | 0.0066 |
| Figure 6A | One way ANOVA with Tukey *post hoc* analysis | Ctrl vs LPS+ATP | <0.0001 |
|  |  | LPS+ATP vs LPS+ATP+THz | <0.0001 |
|  |  | LPS+ATP+THz vs LPS+ATP+THz+siAC9 | 0.0122 |
| Figure 6B | One way ANOVA with Tukey *post hoc* analysis | Ctrl vs LPS+ATP | <0.0001 |
|  |  | LPS+ATP vs LPS+ATP+THz | <0.0001 |
|  |  | LPS+ATP+THz vs LPS+ATP+THz+siAC9 | 0.0003 |
| Figure 6C | One way ANOVA with Tukey *post hoc* analysis | Ctrl vs LPS+ATP | <0.0001 |
|  |  | LPS+ATP vs LPS+ATP+THz | <0.0001 |
|  |  | LPS+ATP+THz vs LPS+ATP+THz+siAC9 | <0.0001 |
| Figure 6D | One way ANOVA with Tukey *post hoc* analysis | Ctrl vs LPS+ATP | <0.0001 |
|  |  | LPS+ATP vs LPS+ATP+THz | <0.0001 |
|  |  | LPS+ATP+THz vs LPS+ATP+THz+siAC9 | <0.0001 |
| Figure 6E | One way ANOVA with Tukey *post hoc* analysis | Ctrl vs LPS+ATP | <0.0001 |
|  |  | LPS+ATP vs LPS+ATP+THz | <0.0001 |
|  |  | LPS+ATP+THz vs LPS+ATP+THz+siAC9 | 0.037 |
| Figure 6G | Unpaired t-test | Ctrl vs THz | 0.9656 |
| Figure 6H | Unpaired t-test | AC9 plasmid vs AC9 plasmid +THz | 0.0043 |
| Figure 7C | Unpaired t-test | Sham vs Sham+THz | 0.8737 |
| Figure 7D | Unpaired t-test | Sham vs Sham+THz | 0.0746 |
| Figure S1A  Distance | One way ANOVA with Bonferroni *post hoc* analysis | Sham vs TBI | 0.0389 |
|  |  | TBI vs TBI+4mW/mm^2^ | >0.9999 |
|  |  | TBI vs TBI+7mW/mm^2^ | 0.4710 |
| Figure S1A  Mean speed | One way ANOVA with Bonferroni *post hoc* analysis | Sham vs TBI | 0.0389 |
|  |  | TBI vs TBI+4mW/mm^2^ | >0.9999 |
|  |  | TBI vs TBI+7mW/mm^2^ | 0.4708 |
| Figure S1B  Center entries | One way ANOVA with Bonferroni *post hoc* analysis | Sham vs TBI | 0.3505 |
|  |  | TBI vs TBI+7mW/mm^2^ | 0.4234 |
| Figure S1B  Time in center | One way ANOVA with Bonferroni *post hoc* analysis | Sham vs TBI | 0.8304 |
|  |  | TBI vs TBI+7mW/mm^2^ | 0.7133 |
| Figure S1B  Total distance | One way ANOVA with Bonferroni *post hoc* analysis | Sham vs TBI | 0.1186 |
|  |  | TBI vs TBI+7mW/mm^2^ | 0.2748 |
| Figure S2B | Unpaired t test | 10 min  TBI vs TBI+THz | 0.4145 |
| Figure S2B | Unpaired t test | 1 h  TBI vs TBI+THz | >0.9999 |
| Figure S2B | Unpaired t test | 1 d  TBI vs TBI+THz | 0.3872 |
| Figure S2B | Unpaired t test | 2 d  TBI vs TBI+THz | 0.2246 |
| Figure S3B | One way ANOVA with Bonferroni *post hoc* analysis | TBI vs Sham | 0.0002 |
|  |  | TBI vs TBI+THz | 0.0784 |
| Figure S3C | Brown-Forsythe and Welch ANOVA tests with Dunnett’s T3 post hoc analysis | TBI vs Sham | 0.0410 |
|  |  | TBI vs TBI+THz | 0.4508 |
| Figure S4B | One way ANOVA with Bonferroni *post hoc* analysis | TBI vs Sham | 0.0001 |
|  |  | TBI vs TBI+THz | >0.9999 |
| Figure S4D | One way ANOVA with Bonferroni *post hoc* analysis | TBI vs Sham | 0.1758 |
|  |  | TBI vs TBI+THz | >0.9999 |
| Figure S4E | One way ANOVA with Bonferroni *post hoc* analysis | TBI vs Sham | 0.0294 |
|  |  | TBI vs TBI+THz | 0.3110 |
| Figure S4G | One way ANOVA with Bonferroni *post hoc* analysis | TBI vs Sham | 0.1558 |
|  |  | TBI vs TBI+THz | 0.2936 |
| Figure S6A | Unpaired t test | TBI vs TBI+THz | 0.0002 |
| Figure S6B | Unpaired t test | TBI vs TBI+THz | 0.0012 |
| Figure S6C | Unpaired t test | TBI vs TBI+THz | 0.0168 |
| Figure S6D | Unpaired t test | TBI vs TBI+THz | 0.0302 |
| Figure S6E | Unpaired t test | TBI vs TBI+THz | 0.0175 |
| Figure S6F | Unpaired t test | TBI vs TBI+THz | 0.0138 |
| Figure S6G | Unpaired t test | TBI vs TBI+THz | 0.0015 |
| Figure S6H | Unpaired t test | TBI vs TBI+THz | 0.0006 |
| Figure S7A | Unpaired t test | TBI vs TBI+THz | 0.0025 |
| Figure S7B | Unpaired t test | TBI vs TBI+THz | 0.0025 |
| Figure S7C | Unpaired t test | TBI vs TBI+THz | 0.0057 |
| Figure S7D | Unpaired t test | TBI vs TBI+THz | 0.0032 |
| Figure S7E | Unpaired t test | TBI vs TBI+THz | 0.1189 |
| Figure S7F | Unpaired t test | TBI vs TBI+THz | 0.0978 |
| Figure S7G | Unpaired t test | TBI vs TBI+THz | 0.002 |
| Figure S7H | Unpaired t test | TBI vs TBI+THz | 0.0029 |
| Figure S8A | Unpaired t test | siNC vs siAC9 | 0.0040 |
| Figure S8B | Unpaired Welch’s t-test | siNC vs siAC9 | 0.0009 |

**Supplementary Methods**

**Open field test**

The open field test was performed to evaluate spontaneous locomotor activity and anxiety‑like behavior. Each mouse was placed in the center of the experimental arena and allowed to explore freely for 10 minutes. The arena was divided into a central zone and a peripheral zone. Animal behavior was recorded by an overhead video camera, and the following parameters were analyzed using Smart analysis software (v3.0). The apparatus was thoroughly cleaned with 70% ethanol between trials to eliminate olfactory cues.

**Blood flow imaging**

The RFLSI Ⅲ/Ⅲ-SE laser speckle flow imaging system was used for blood flow imaging. Following THz photons exposure, the imaging probe was vertically positioned above the cranial window of mice, and the field of view (FOV) was adjusted to ensure full coverage of the injured brain region. The imaging parameters were optimized and fixed as follows: frame rate = 30 frames per second, exposure time = 10 ms, and imaging duration = 5 minutes per session. All speckle images were analyzed using the system’s proprietary software, which converted speckle contrast into relative cerebral blood flow (CBF) values, expressed as perfusion units (PU).
